# SIRT1 selectively exerts the metabolic protective effects of hepatocyte nicotinamide phosphoribosyltransferase

**DOI:** 10.1038/s41467-022-28717-7

**Published:** 2022-02-28

**Authors:** Cassandra B. Higgins, Allyson L. Mayer, Yiming Zhang, Michael Franczyk, Samuel Ballentine, Jun Yoshino, Brian J. DeBosch

**Affiliations:** 1 Department of Pediatrics, Washington University School of Medicine, St. Louis, MO 63110 USA; 2 Biogenerator, St. Louis, MO 63110 USA; 3 Department of Medicine, Keio University School of Medicine, Minato, Tokyo, Japan; 4 Department of Anatomic and Molecular Pathology, Washington University School of Medicine, St. Louis, MO 63110 USA; 5 Department of Cell Biology & Physiology, Washington University School of Medicine, St. Louis, MO 63110 USA

**Keywords:** Cell signalling, Diabetes, Dyslipidaemias, Hepatology

## Abstract

Calorie restriction abates aging and cardiometabolic disease by activating metabolic signaling pathways, including nicotinamide adenine dinucleotide (NAD^+^) biosynthesis and salvage. Nicotinamide phosphoribosyltransferase (NAMPT) is rate-limiting in NAD^+^ salvage, yet hepatocyte NAMPT actions during fasting and metabolic duress remain unclear. We demonstrate that hepatocyte NAMPT is upregulated in fasting mice, and in isolated hepatocytes subjected to nutrient withdrawal. Mice lacking hepatocyte NAMPT exhibit defective FGF21 activation and thermal regulation during fasting, and are sensitized to diet-induced glucose intolerance. Hepatocyte NAMPT overexpression induced FGF21 and adipose browning, improved glucose homeostasis, and attenuated dyslipidemia in obese mice. Hepatocyte SIRT1 deletion reversed hepatocyte NAMPT effects on dark-cycle thermogenesis, and hepatic FGF21 expression, but SIRT1 was dispensable for NAMPT insulin-sensitizing, anti-dyslipidemic, and light-cycle thermogenic effects. Hepatocyte NAMPT thus conveys key aspects of the fasting response, which selectively dissociate through hepatocyte SIRT1. Modulating hepatocyte NAD^+^ is thus a potential mechanism through which to attenuate fasting-responsive disease.

## Abstract (web summary)

NAD + metabolism is potential target to treat metabolic disorders, in part due to the effects of the NAD + dependent enzyme Sirt1. Here the authors report that hepatic nicotinamide phosphoribosyltransferase, a rate-limiting step in the NAD + salvage pathway, regulates dark-cycle thermogenesis in a Sirt1-dependent but light-cycle thermogenesis and glucose homeostasis in a Sirt1-independent manner.

## Introduction

Intermittent fasting and caloric restriction improve metabolic and inflammatory parameters in mice and humans, and both are promising means by which to abate diseases of aging, overnutrition, and neurodegeneration^1–4^. Positioned at the nexus of portal and venous circulations, where an organism’s current and impending glycemic states are first encountered, the hepatocyte is uniquely positioned to coordinate the transition between fed and fasting metabolism. Accordingly, interdicting hepatocyte glucose transport, or simply activating its central downstream signaling pathways, is sufficient to mimic key aspects of the therapeutic hepatocyte fasting response^5–16^.

Fasting and caloric restriction in mammals activate the sirtuins, which are mammalian homologues of the stress-responsive protein deacetylase in yeast, *sir2*^17,18^. In response to exercise, lean diet and fasting, mammalian sirtuins adaptively regulate transcriptional, enzymatic, inflammatory, and circadian processes^19–23^. Hepatocyte SIRT1 has recently emerged as a critical regulator of hepatic and peripheral glucose and energy homeostasis^19,24–30^, largely in part due to its downstream transcriptional regulation of the anti-diabetic hepatokine, fibroblast growth factor 21 (FGF21^28,31,32^). Several groups have thus pursued exogenous means by which to activate SIRT1, including through administering resveratrol, allosteric regulation, or by treating with precursors to the obligate SIRT1 cofactor, nicotinamide dinucleotide (NAD^+^^33–40^). The preponderance of current data underscore the importance of the adipocyte as the precursor target tissue for NAD^+^ biosynthesis, and the central hub of inter-tissue communication^25,41–44^.

NAD^+^ is derived from multiple sources:^44,45^ exogenous dietary input, endogenous de novo biosynthesis, and through the NAD^+^ salvage pathway. Among the most proximal NAD^+^ intermediaries is nicotinamide mononucleotide (NMN^46^), the synthesis of which is catalyzed by nicotinamide phosphoribosyltransferase (NAMPT). This NAMPT-mediated reaction is a rate-limiting step in the NAD^+^ salvage pathway in mammals. Nevertheless, the specific function of hepatocyte NAD^+^ salvage in contrast with other NAD^+^ biosynthetic pathways, and the targeted contributions of the hepatocyte in driving this pathway during fasting and metabolic stress are not yet fully appreciated.

Here, we demonstrate that hepatocyte NAMPT mediates a key hepatocyte signaling cascade during fasting. We provide data that fasting and hepatocyte glucose transport inhibition upregulate hepatocyte NAMPT. Mice lacking hepatocyte NAMPT failed to properly regulate fasting-induced FGF21 expression and thermal homeostasis. Hepatocyte-specific NAMPT overexpression enhanced hepatic FGF21 expression, peripheral thermogenesis, and glucose homeostasis in diet-induced and genetically obese models. Strikingly, in diet-induced obese models, hepatocyte SIRT1 was selectively required for NAMPT-mediated effects on FGF21 secretion, and dark-cycle thermogenesis. In contrast, hepatocyte SIRT1 was dispensable for NAMPT to produce therapeutic effects on light-cycle thermogenesis, dyslipidemia, and hepatic de novo lipogenesis. We conclude that hepatocyte NAMPT exerts broad fasting-mimetic effects downstream of generalized fasting and hepatocyte glucose transport inhibition, and that its canonical effector deacetylase, SIRT1, selectively mediates these metabolic adaptations. Interdicting metabolic disease at the level of NAD^+^ biosynthesis and/or hepatocyte glucose transport are proximal targets that may leverage the broader effects of hepatocyte NAMPT activation against aging, obesity, and other fasting-responsive diseases.

## Results

### Fasting and glucose transport inhibition activate hepatocyte NAMPT

**Fig. 1:**
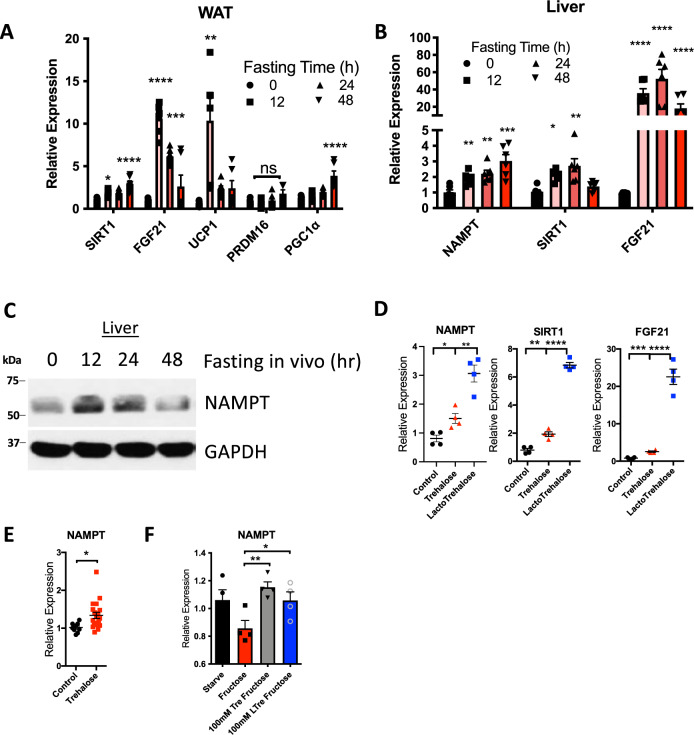
Fasting and GLUT blockade upregulated hepatocyte NAMPT. **A** Browning marker gene quantification by qPCR in epididymal white adipose tissue (WAT) from WT mice fasted 0–48 h. *n* = 6 mice for each group. Circles, 0 h fast; squares, 12 h fast; upward triangle, 24 h; downward triangle, 48 h. **B** NAMPT, SIRT1, and FGF21 gene expression in livers from WT mice fasted 0–48 h. *n* = 6 mice for each group. Symbols: Circles, fasting 0 h. Squares, 12 h. Upward triangles, 24 h, downward triangle, 48 h. **C** Representative (from *n* = 4 per group) NAMPT immunoblot in livers from WT mice fasted 0–48 h. **D** qPCR quantification of NAMPT, SIRT1, FGF21 gene expression in primary hepatocytes treated with the fasting-mimetic glucose transporter inhibitors, trehalose and lactotrehalose. *n* = 4 per group. Circles, cultures treated with regular growth media, Red triangles, trehalose-treated, Blue squares, LactoTrehalose-treated. **E** qPCR in liver from WT mice treated with 5-day 3% trehalose ad libitum in drinking water. *n* = 8 vehicle- (black circles) and 18 trehalose-treated (red squares). **F** Fasting-inducible NAMPT expression by qPCR in isolated primary hepatocytes treated with or without 10 mM fructose in the presence or absence of carbohydrate transporter inhibitors trehalose or lactotrehalose. *n* = 3 starved, 4 fructose-treated, 4 trehalose- and fructose-treated, 4 lactotrehalose- and fructose-treated. Circles, cultures treated with regular growth media; squares, fructose-treated; downward triangles, cultures treated with trehalose and fructose; open circles, LactoTrehalose and fructose-treated cultures. Error bars in (**A**–**B**), (**D**–**F**) represent standard error of the mean (SEM). *P < 0.05, **P < 0.01, ***P < 0.001, ****P < 0.0001. Statistical tests: (**A**–**F**), two-tailed *T*-test, Bonferroni–Dunn post hoc.

Fasting in hibernating mammals is characterized by oscillatory hypothermia and transient hyperthermia^47^. This is mediated by increased UCP1 content and mitochondrial proliferation in white adipose tissue^47^. We therefore assessed the effects of fasting on epididymal white adipose tissue (eWAT) browning marker gene expression in wild-type C57B6/J mice (Fig. 1A). Uncoupling protein-1 and PPARγ-coactivator-1α (PGC1α) were upregulated during a 12–48 h fast. This was associated with expression changes in previously described WAT signaling pathways that drive this browning pathway. Specifically, expression of the protein deacetylase, SIRT1, and FGF21 were each increased in eWAT during 12–48 h fasting (Fig. 1A).

WAT SIRT1 activation here and in prior reports^19,43,48,49^ suggested that fasting enhances NAD^+^ metabolism. The hepatocyte is a key driver of energy metabolism during generalized fasting and upon glucose-specific deprivation^1,5–7,9–11,50–52^. Therefore, we examined whether hepatocyte-adipose browning signaling pathways associated with increases in NAD^+^ salvage via the NAMPT pathway. Hepatic FGF21, SIRT1, and NAMPT expression increased within 24 h fasting (Fig. 1B). Immunoblot data confirmed that increased hepatic NAMPT expression accompanied increased NAMPT protein abundance during fasting (Fig. 1C).

We identified trehalose and its degradation-resistant trehalose analogue, lactotrehalose (LT), as fasting-mimetic compounds that activate the hepatocyte fasting-like response^5,6,8,9,53,54^. These compounds activate fasting-like signaling pathways, in part, by inhibiting glucose transport into the hepatocyte. We asked if trehalose and LT^6,53–55^ also induced NAMPT expression in isolated primary murine hepatocytes incubated in standard growth media (10% FCS, 4.5 g/L glucose). Analogous to the effect of physiological fasting in mice, the hepatocyte glucose transporter inhibitors, trehalose and LT, induced FGF21, NAMPT and UCP1 expression (Fig. 1D). Similarly, in vivo 24 h oral trehalose feeding (3% in drinking water, ad libitum) modestly induced liver NAMPT expression (Fig. 1E).

We next assessed the ability of trehalose and LT to reverse fructose-induced suppression of hepatocyte fasting responses in vitro. To that end, WT primary murine hepatocytes were treated in starve media (0.5% FCS, 1 g/L glucose) with or without 10 mM fructose in the presence or absence trehalose or LT (Fig. 1F). Fructose incubation suppressed fasting-induced NAMPT expression in hepatocytes, whereas trehalose and LT reversed fructose-induced NAMPT suppression (Fig. 1F). Therefore, fasting, trehalose and LT cell-autonomously induce hepatic NAMPT expression in vitro and in vivo models.

### Hepatocyte NAMPT mediates fasting thermogenesis and fasting-induced hepatic FGF21

**Fig. 2:**
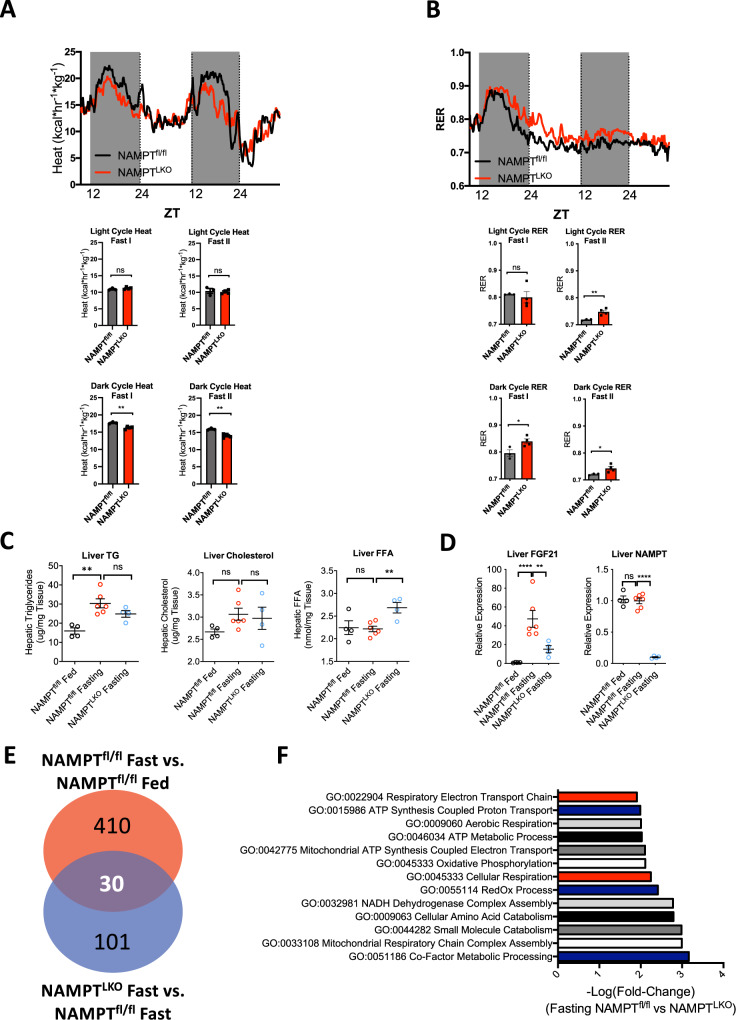
Mild fasting metabolic defects in NAMPT^LKO^ mice. **A** Heat-Zeitgeber Time tracing in WT and NAMPT^LKO^ littermate mice during light and dark cycles during fasting. Mean heat production in WT and NAMPT^LKO^ mice during light and dark cycle fed and early (Fasting I, 1- 24 h) and prolonged (Fasting II, 24–48 h) fasting periods is graphed below. *n* = 3 WT, 4 NAMPT^LKO^ mice. **B** RER-Zeitgeber time tracing in WT and NAMPT^LKO^ littermate mice during light and dark cycles during ad-lib fed (first 24 h) and fasting periods. Mean RER in WT and NAMPT^LKO^ mice during light and dark cycle fed and fasting periods is graphed below. *n* = 3 WT, 4 NAMPT^LKO^ mice. Error bars in (**A**–**B**) (bottom panels) represent SEM. Circles, WT mice. Squares, NAMPT^LKO^ mice for (**A** and **B**). **C** Liver triglycerides (TG), total cholesterol and non-esterified fatty acid (FFA) quantification following 48 h fasting in WT and NAMPT^LKO^ mice. *n* = 4 WT Fed, 6 WT Fasting; 4 NAMPT^LKO^ Fasting. **D** qPCR quantification of liver FGF21, NAMPT in fed and 48 h-fasting WT and NAMPT^LKO^ mice. *n* = 4 WT Fed, 6 WT Fasting; 4 NAMPT^LKO^ Fasting. Error bars in (**C** and **D**). represent SEM. Black circles, Fed WT, Red circles, fasting WT, Blue circles, Fasting NAMPT^LKO^ mice. **E** Venn diagram representing significantly altered genes (*P* < 0.05) in random-fed vs. 12 h fasting WT and NAMPT^LKO^ mice, quantified by unbiased transcriptomics. *n* = 2 WT Fed, 4 WT Fasting; 5 NAMPT^LKO^ Fasting. **F** Bar plot demonstrating −log(FC) for significantly down-regulated gene ontology (GO) pathways revealed when comparing WT vs. NAMPT^LKO^ livers (*P* < 0.05). *n* = 4 WT Fed, 6 WT Fasting; 4 NAMPT^LKO^ Fasting. Statistical tests: (**A**) (upper panel), ANCOVA. **A** (lower panels), (**B**–**D**), two-tailed *T*-test, Bonferroni–Dunn post hoc correction. **E**, **F** EdgeR Exact, Benjamini–Hochberg post hoc correction.

To test the hypothesis that hepatocyte NAMPT is required for appropriate transcriptional and thermic responses to fasting, we subjected WT and NAMPT^LKO^ littermates to indirect calorimetry during acute and prolonged fasting (1–24 h and 24–48 h fasting) protocols. We first confirmed reduced hepatic [NAD^+^] by high-performance liquid chromatography in WT and NAMPT-deficient livers (Supplementary Fig. 1A). During the light cycle of each 24 h period, NAMPT^LKO^ and WT mice had equivalent thermal regulation, regardless of fed or fasting status (Fig. 2A). In contrast, dark cycle heat generation was impaired in NAMPT^LKO^ mice only during the fasting period (Fig. 2A). Light and dark cycle respiratory exchange ratio (RER) in NAMPT^LKO^ mice was significantly greater throughout fasting light and dark cycles (Fig. 2B). We confirmed that these relationships were not biased toward prolonged fasting effects by sub-analysis of mean heat generation in each group over the course of 1 h after 6 h total fasting (Supplementary Fig. 1B). This revealed identical statistical relationships between WT and NAMPT^LKO^ heat and RER during acute fasting after 6 h fasting, vs. prolonged fasting periods up to 12–48 h. Liver TG and cholesterol accumulation was not affected in fasting NAMPT^LKO^ mice relative to fasting WT mice (Fig. 2C), although fasting levels of non-esterified fatty acids was increased in fasting NAMPT^LKO^ mice vs. fasting WT mice (Fig. 2C). However, fasting-induced hepatic FGF21 mRNA induction was significantly blunted in NAMPT^LKO^ mice Fig. 2D). To more broadly address physiological hepatocyte NAMPT fasting functions, we examined livers from NAMPT^fl/fl^ and NAMPT^LKO^ mice after 12 h fasting by unbiased transcriptomics (Fig. 2E). This revealed 440 significantly altered genes in fasting vs. fed NAMPT^fl/fl^ liver (Fig. 2E, in red). 131 hepatic transcripts were significantly altered in fasting NAMPT^LKO^ mice, vs. fasting NAMPT^fl/fl^ mice (Fig. 2E, in blue). Of these differentially expressed genes, 30 were differentially expressed in both comparisons (Fig. 2E, overlap). Gene Ontology analysis and confirmatory CompBio analysis revealed that hepatocyte-specific NAMPT deficiency impaired several key hepatic fasting metabolic processes. Among the most significantly impaired hepatic Gene Ontology processes in fasting NAMPT^LKO^ mice were cofactor and redox metabolism, ATP synthesis and electron transport, carbon and amino acid catabolism, and oxidative phosphorylation (Fig. 2F) without significant changes in ketone body biosynthesis. The data are consistent with the hypothesis that NAMPT is required to regulate hepatocyte redox and bioenergetic physiology during fasting.

### Hepatocyte NAMPT protects against diet-induced glucose intolerance

**Fig. 3:**
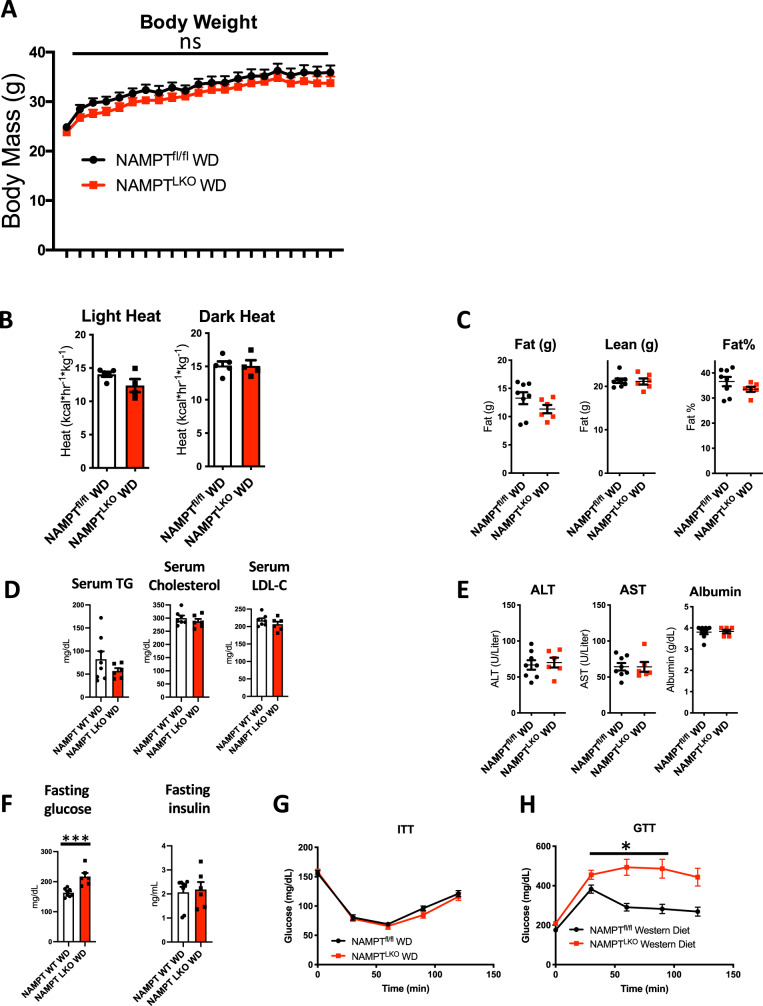
Hepatocyte NAMPT protects against diet-induced glucose intolerance. **A** Body weights over time in WT and NAMPT^LKO^ mice on 12wk Western diet. *n* = 8 WT, 6 NAMPT^LKO^ mice. **B** WT and NAMPT^LKO^ mice were subjected to 12wk Western dietary feeding and metabolic phenotyping by indirect calorimetry at 4wk and 12wk post-initiation of dietary intervention. Mean heat generation during light and dark cycles is demonstrated for light and dark cycles. *n* = 5 WT, 4 NAMPT^LKO^ mice. **C** Fat and lean mass and % body fat in WD-fed WT and NAMPT^LKO^ mice. *n* = 8 WT, 6 NAMPT^LKO^ mice. **D** Circulating lipids in 12wk WD-fed WT and NAMPT^LKO^ mice. *n* = 8 WT, 6 NAMPT^LKO^ mice. TG, triglycerides; LDL-C, low-density lipoprotein cholesterol. **E** Serum transaminases, albumin and liver weight-to-body weight ratios in 12wk WD-fed WT and NAMPT^LKO^ mice. *n* = 8 WT, 6 NAMPT^LKO^ mice. **F** 4 h fasting insulin and glucose in 12wk WD-fed WT and NAMPT^LKO^ mice. *n* = 8 WT, 6 NAMPT^LKO^ mice. **G** and **H** Insulin tolerance and glucose tolerance testing in WT and LKO mice after 12wk WD feeding. *n* = 8 WT, 6 NAMPT^LKO^ mice. *P < 0.05 vs. NAMPT WT. Error bars in (**A**–**F** and **H**) represent SEM. Circles, Western diet-fed WT, Squares, Western diet-fed NAMPT^LKO^. Statistical tests: (**A**, **G**, **H**), repeated measures ANOVA. **B**–**F** Two-tailed *T*-test.

We next subjected NAMPT^fl/fl^ (WT) and NAMPT^LKO^ mice to 12wk Western diet feeding to test the hypothesis that hepatocyte NAMPT protects against the deleterious metabolic effects of overnutrition. We selected this model of diet-induced metabolic disease in part because it models both the high fat and carbohydrate content that is prevalent in the diets of modern industrialized populations^56^. Throughout its 12wk dietary challenge, body weight (Fig. 3A) and caloric expenditure during light- and dark cycles (Fig. 3B) were unaffected in NAMPT^LKO^ mice. Similarly, total body fat mass, lean mass, and circulating TG, total cholesterol and LDL were unaffected by hepatocyte NAMPT deficiency (Fig. 3C, D). Similarly, liver weight-to-body weight ratio, transaminases and albumin were unchanged in NAMPT^LKO^ mice (Fig. 3E). In contrast, fasting glucose (16 h) was significantly elevated in NAMPT^LKO^ mice when compared with WT mice, whereas plasma insulin was not statistically different (Fig. 3F). Accordingly, insulin tolerance (Fig. 3G) was unaltered in NAMPT^LKO^ mice relative to WT mice, whereas NAMPT^LKO^ mice were significantly more glucose intolerant (Fig. 3H). Overall, loss-of-function data suggest that hepatocyte NAMPT is essential to protect against diet-induced glucose intolerance.

### Increased hepatocyte [NAD^+^] links NAMPT overexpression with increased hepatic FGF21 expression

These findings led us to define the consequences of direct NAMPT upregulation in hepatocytes. FGF21 improves peripheral glucose homeostasis in mice and in humans^31^. Therefore, we first asked whether hepatocyte NAMPT cell-autonomously induces FGF21 expression. Primary murine hepatocytes transfected with adenovirus encoding wild-type murine NAMPT increased SIRT1, PGC1α, and FGF21 expression when compared with GFP-transfected hepatocytes. Pan-FGF receptor inhibitor, LY2874455 (FGFRi) had no suppressive effect on gene expression for NAMPT or for any of the signaling intermediaries quantified (Supplementary Fig. 2A), suggesting that autocrine FGF receptor function is not required for NAMPT regulation of SIRT1, FGF21, and PGC1α. However, an inhibitor of exocytosis, brefeldin A, blocked appearance of the FGF21 peptide in the extracellular media of isolated hepatocytes overexpressing NAMPT (Supplementary Fig. 2B). Similarly, brefeldin A increased NAMPT protein, but not mRNA, in the hepatocyte lysate fraction in treated cultures overexpressing NAMPT (Supplementary Fig. 2B and 2C). In contrast, in unperturbed primary murine hepatocytes, NAMPT overexpression also induced FGF21 (Supplementary Fig. 2D). The data suggest that NAMPT activates hepatocyte FGF21 expression and secretion. To interrogate this pathway in vivo, we expressed GFP or NAMPT in WT C57B6/J mice by tail-vein adenovirus. AdNAMPT increased both hepatic [NAD^+^] and FGF21 protein and mRNA 48 h after AdNAMPT delivery in vivo (Supplementary Fig. 3A, C). This was associated with increased NAD^+^-dependent lysine deacetylase activity in cultured AML12 hepatocytes (Supplementary Fig. 3B). In this acute phase, hepatic triglycerides, hepatic cholesterol and caloric expenditure were not altered in lean mice overexpressing hepatic NAMPT (Supplementary Fig. 3D, E).

### Hepatic NAMPT induces hepatic FGF21 and enhances glucose homeostasis in leptin receptor-deficient obese mice

**Fig. 4:**
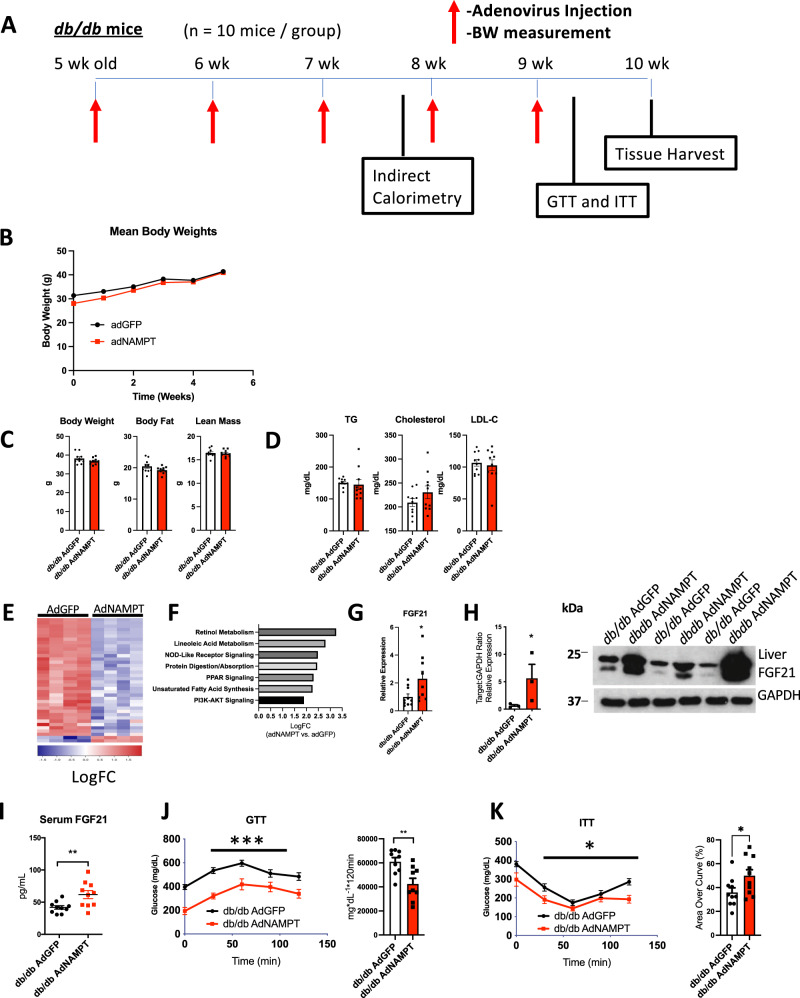
NAMPT induces FGF21 and insulin sensitivity independent of lepR signaling. **A** Experimental design for *db/db* AdNAMPT analyses. *n* = 10 *db/db* AdGFP; 10 *db/db* AdNAMPT mice. Circles, *db/db* AdGFP mice, Squares *db/db* AdNAMPT mice. **B** Body weight over time in *db/db* mice expressing GFP or NAMPT. *n* = 10 *db/db* AdGFP; 10 *db/db* AdNAMPT mice. Circles, *db/db* AdGFP mice, Squares *db/db* AdNAMPT mice. **C** Body composition by echoMRI analysis in mice expressing GFP or NAMPT. *n* = 10 *db/db* AdGFP; 10 *db/db* AdNAMPT mice. Circles, *db/db* AdGFP mice, Squares *db/db* AdNAMPT mice. **D** Serum triglycerides (TG), cholesterol and low-density lipoprotein cholesterol (LDL-C) in *db/db* mice treated with adenovirus to overexpress GFP or NAMPT. *n* = 10 *db/db* AdGFP; 10 *db/db* AdNAMPT mice. Circles, *db/db* AdGFP mice, Squares *db/db* AdNAMPT mice. **E** RNAseq quantification heatmap, arranged by calculated hierarchical clustering, demonstrating genes *P* < 0.05 in *db/db* AdNAMPT vs. AdGFP mice. *n* = 4 *db/db* AdGFP; 4 *db/db* AdNAMPT mice. Color scale represents Log(FC) (**F**). Most significantly altered KEGG pathways demonstrating log(FC) between *db/db* AdNAMPT and *db/db* AdGFP livers. *n* = 10 *db/db* AdGFP; 10 *db/db* AdNAMPT mice. Unadjusted *P* < 0.05 for all pathways shown. **G** qPCR confirmation of RNAseq data suggesting upregulated liver FGF21 for *n* = 10 *db/db* AdGFP; 9 *db/db* AdNAMPT mice. Circles, *db/db* AdGFP mice, Squares *db/db* AdNAMPT mice. **H** and **I** FGF21 quantification by immunoblot analysis and serum ELISA in *db/db* AdGFP and *db/db* AdNAMPT mice. *n* = 3 *db/db* AdGFP; 3 *db/db* AdNAMPT mice for immunoblot data. *n* = 10 *db/db* AdGFP; 10 *db/db* AdNAMPT mice for ELISA data. *P < 0.05, **P < 0.01 vs. AdGFP. **J** Glucose tolerance testing and area under the GTT curve in *db/db* mice treated with adenovirus encoding GFP or NAMPT. *n* = 9 *db/db* AdGFP; 9 *db/db* AdNAMPT biologically independent mice. **K** Insulin tolerance testing and % area over the ITT curve in *db/db* mice treated with adenovirus encoding GFP or NAMPT. *n* = 10 *db/db* AdGFP; 10 *db/db* AdNAMPT mice. In (**G**–**K**), Circles, *db/db* AdGFP mice, Squares *db/db* AdNAMPT mice. *P < 0.05, **P < 0.01, ***P < 0.001, ****P < 0.0001. Error bars in (**G**, **H**) (left), (**I**–**K**) represent SEM. Statistical tests: (**B**, **J**, **K**), repeated measures ANOVA. **C**, **D**, **G**, **H**, **I**, **J** (right panel), (**K**) (right panel) two-tailed *T*-test. **F** EdgeR Exact, Benjamini–Hochberg post hoc correction.

We next tested the hypothesis that activating hepatocyte NAMPT attenuates glucose intolerance in diabetic models (Fig. 4A). *LepR*-deficient (*db/db*) mice overexpressing NAMPT did not regulate body weight, body composition, or circulating lipids differently than GFP-overexpressing mice (Fig. 4B, C, D). However, *db/db* mice overexpressing NAMPT exhibited broad transcriptomic differences as quantified by RNA sequencing in livers obtained from NAMPT- and GFP-overexpressing *db/db* mice (Fig. 4E). We observed highest-magnitude changes in several pathways (significance threshold *P* < 0.05), including NOD-like receptor signaling (e.g., a KEGG-catalogued pathway that encompasses the *Nampt* gene), protein degradation, PPAR signaling, fatty acid synthesis and metabolism, and insulin signaling through the PI3K-Akt pathway (Fig. 4F). For example, FGF21 was significantly upregulated, confirmed by qRT-PCR in crude liver cDNA preparations from *db/db* x AdGFP and *db/db* x AdNAMPT overexpressing mice (Fig. 4G). NAMPT overexpression similarly increased liver FGF21 protein (Fig. 4H) and FGF21 peptide in *db/db* liver and serum (Fig. 4I), when compared with GFP-overexpressing *db/db* mice. These transcriptomic hallmarks of hepatic insulin sensitivity, and increased hepatic and circulating FGF21 were accompanied by significantly lower plasma glucose throughout both glucose and insulin tolerance testing curves in NAMPT-expressing *db/db* mice (Fig. 4J, K and Supplementary Fig. 4A).

### NAMPT enhances energy homeostasis in leptin receptor-deficient obese mice

**Fig. 5:**
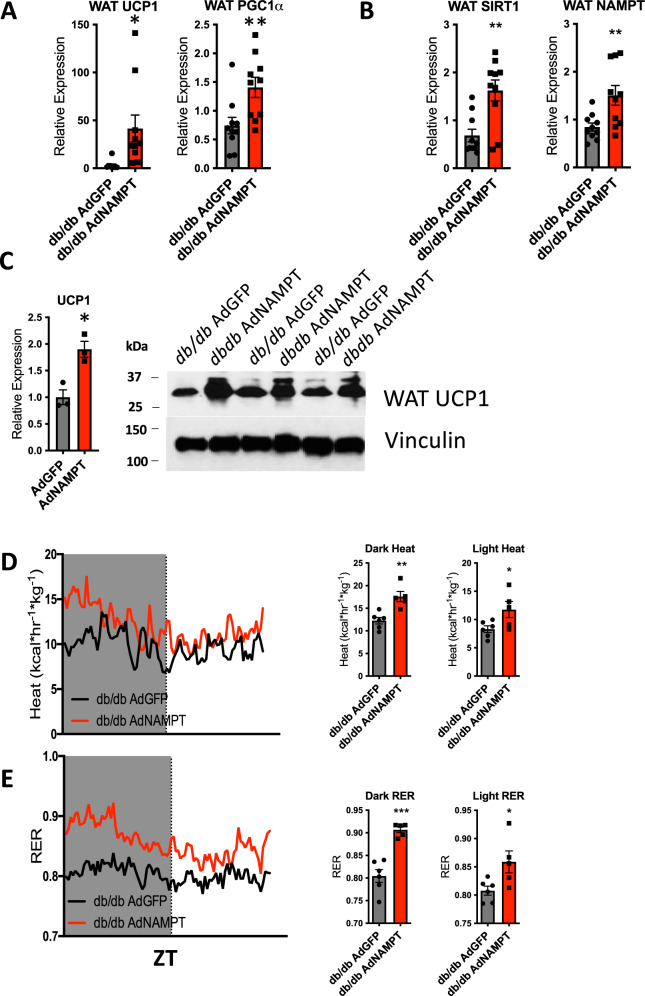
NAMPT overexpression induces white adipose tissue (WAT) browning and thermogenesis in *db/db* mice. **A** and **B** qPCR analysis of white adipose tissue UCP1, PGC1α, NAMPT, and SIRT1 expression in mice 7 days after tail-vein delivery of adenovirus encoding *db/db* AdGFP or *db/db* AdNAMPT. *n* = 10 *db/db* AdGFP; 10 *db/db* AdNAMPT mice. **C** Representative immunoblot analysis of WAT UCP1 protein accumulation from *n* = 3 *db/db* AdGFP and *n* = 3 *db/db* AdNAMPT mice. **D** Left, heat-ZT tracing in GFP- and NAMPT-overexpressing *db/db* mice. *n* = 6 *db/db* AdGFP; 5 *db/db* AdNAMPT mice. Right, quantification of mean heat by indirect calorimetry during dark and light cycles. **E** Left, RER-ZT tracing in GFP- and NAMPT-overexpressing *db/db* mice. *n* = 6 *db/db* AdGFP; 5 *db/db* AdNAMPT mice. Quantification of mean RER by indirect calorimetry in *db/db* AdGFP and *db/db* AdNAMPT mice during light and dark cycles. *n* = 6 *db/db* AdGFP; 5 *db/db* AdNAMPT mice. Circles, *db/db* AdGFP mice, Squares *db/db* AdNAMPT mice. *P < 0.05, **P < 0.01, ***P < 0.001 vs. bracketed control. Error bars in (**A**–**C**) (left), and (**E**). represent SEM. Statistical tests: (**A**–**C**), two-tailed *T*-test. **D**, **E** ANCOVA.

To test the hypothesis that hepatic NAMPT regulates peripheral adipose browning and thermogenesis, we analyzed the effects of hepatocyte-specific NAMPT overexpression on WAT browning markers UCP1 and PGC1α. Both marker genes were induced by NAMPT adenoviral expression (Fig. 5A), as were WAT SIRT1 and NAMPT both in NAMPT-overexpressing *db/db* mice in vivo (Fig. 5B), and in FGF21-treated adipose tissue explants (Supplementary Fig. 4B). UCP1 protein was similarly increased in WAT crude lysates obtained from NAMPT-overexpressing *db/db* mice (Fig. 5C) when compared with GFP-expressing mice.

To define the functional outcome of these molecular changes in the WAT depot, we subjected *db/db* x AdNAMPT and *db/db* x AdGFP mice to indirect calorimetry. AdNAMPT-treated mice exhibited significantly greater light and dark cycle thermogenesis, O_2_ and CO_2_ exchange, and RER, when compared with GFP-overexpressing mice (Fig. 5D, E). Taken together, this demonstrates that hepatocyte NAMPT expression induces hepatic *fgf21* expression, and improves thermogenesis, and peripheral glucose homeostasis in diabetic mice, independent of leptin receptor signaling.

### SIRT1 distinguishes NAMPT-mediated control of light- and dark-cycle energy utilization

**Fig. 6:**
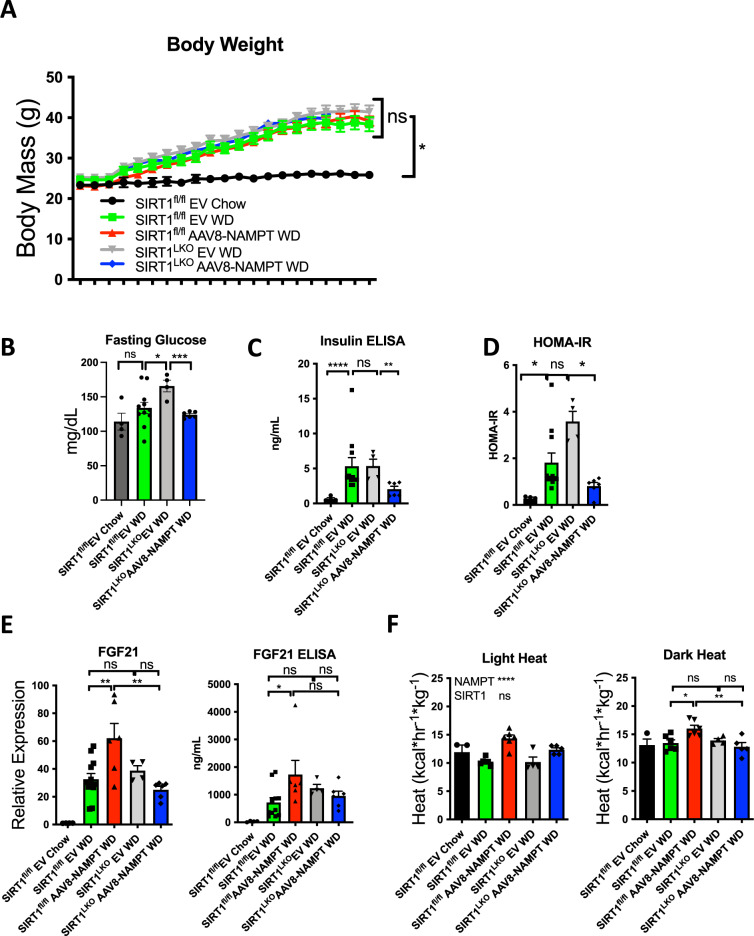
NAMPT induces SIRT1-dependent hepatic FGF21 and selectively enhances energy metabolism. **A** Body weights over time in WT and SIRT1^LKO^ mice overexpressing empty vector or NAMPT on 12wk Western diet. *n* = 3 SIRT1^fl/fl^ Chow; 9 SIRT1^fl/fl^ EV WD; 6 SIRT1^fl/fl^ AAV8-NAMPT WD; 5 SIRT1^LKO^ EV WD. **B**–**D** 4 h fasting glucose, insulin, and HOMA-IR (homeostatic model of insulin resistance − fasting glucose (mg/dL) × fasting insulin (ng/mL)/405) in WT and SIRT1^LKO^ mice overexpressing vector or NAMPT after 12wk chow or Western diet feeding. *n* = 4 SIRT1^fl/fl^ Chow; 11 SIRT1^fl/fl^ EV WD; 4 SIRT1^LKO^ EV WD; 6 SIRT1^LKO^ AAV8-NAMPT WD. **E** FGF21 mRNA and serum peptide, as quantified by qRT-PCR in livers and by serum ELISA in WT and SIRT1^LKO^ mice overexpressing empty vector or NAMPT after Western diet feeding. Interaction, *P* < 0.01 and <0.05 for FGF21 mRNA and peptide, respectively. *n* = 4 SIRT1^fl/fl^ Chow; 11 SIRT1^fl/fl^ EV WD; 6 SIRT1^fl/fl^ AAV8-NAMPT WD; 4 SIRT1^LKO^ EV WD; 6 SIRT1^LKO^ AAV8-NAMPT WD (**F**). Indirect calorimetry in WT and SIRT1^LKO^ mice overexpressing vector or NAMPT after 12wk chow or Western diet feeding. *n* = 3 SIRT1^fl/fl^ Chow; 6 SIRT1^fl/fl^ EV WD; 6 SIRT1^fl/fl^ AAV8-NAMPT WD; 4 SIRT1^LKO^ EV WD; 5 SIRT1^LKO^ AAV8-NAMPT WD. Interactions for light and dark cycle heat: *P* = ns, and *P* < 0.01 respectively. *, **, *P* < 0.05, <0.01 vs. bracketed control by Tukey’s multiple comparisons testing. Error bars in (**A**–**F**) represent SEM. Circles, SIRT1^fl/fl^ Chow; Squares, SIRT1^fl/fl^ EV WD; Upward Triangles SIRT1^fl/fl^ AAV8-NAMPT WD; Downward Triangles, SIRT1^LKO^ EV WD; Diamonds SIRT1^LKO^ AAV8-NAMPT WD. Statistical tests: (**A**), repeated measures ANOVA. **B**–**D** one-way ANOVA with Dunnett’s post hoc testing. **E**, **F** Two-way ANOVA, Tukey’s post hoc correction.

NAMPT induces FGF21 and reverses metabolic derangements in diet-induced and genetic models of obesity and glucose intolerance. We next directly tested the hypothesis that hepatocyte SIRT mediates the therapeutic effects of NAMPT on *fgf21* expression and glucose homeostasis. We subjected SIRT1^fl/fl^ (WT) and hepatocyte-specific SIRT1-deficient (SIRT1^LKO^) littermate mice to chow or Western diet feeding (12wk) in the presence or absence of hepatocyte-specific (thyroxine binding globulin promoter-driven) NAMPT overexpression. Western diet feeding increased body weight gain, and impaired glucose homeostasis wild-type mice (Fig. 6A–C). Although hepatocyte NAMPT expression had no effect on body weight, NAMPT overexpression lowered 4 h fasting glucose, insulin and HOMA-IR in hepatocyte SIRT1-deficient mice (Fig. 6B–D), relative to Western diet-fed, vector-expressing SIRT^LKO^ controls (Fig. 6D).

NAMPT induces *fgf21* expression, protein, and peptide in diabetic mouse livers and peripheral circulation. We therefore tested the hypothesis that hepatocyte NAMPT induces hepatocyte *fgf21* by activating SIRT1 using Western diet-fed WT and SIRT1^LKO^ mice expressing control vector or NAMPT. Again, hepatocyte NAMPT increased hepatic and circulating *fgf21* mRNA and peptide in Western diet-fed WT mice. NAMPT-induced FGF21 expression was abrogated in SIRT1^LKO^ mice, and NAMPT-induced circulating FGF21 peptide trended lower in SIRT1^LKO^ mice vs. WT mice overexpressing hepatocyte NAMPT (Fig. 6E).

Indirect calorimetry in Western diet-fed WT and SIRT1^LKO^ mice revealed that hepatocyte NAMPT overexpression induced heat production in WT mice during both light- and dark-cycles (Fig. 6F). However, hepatocyte SIRT1 deficiency abrogated dark-cycle, but not light-cycle thermogenesis in NAMPT-overexpressing mice (Fig. 6F). This phenocopies the specific dark-cycle thermic defects observed in fasting NAMPT^LKO^ mice (Fig. 2A) and is underscored by significant two-way NAMPT-SIRT1 interactions identified in dark-, but not light-cycle thermogenesis (Fig. 6F). The data indicate that hepatocyte NAMPT-SIRT1 signaling distinguishes light- and dark-cycle energetic control.

**Fig. 7:**
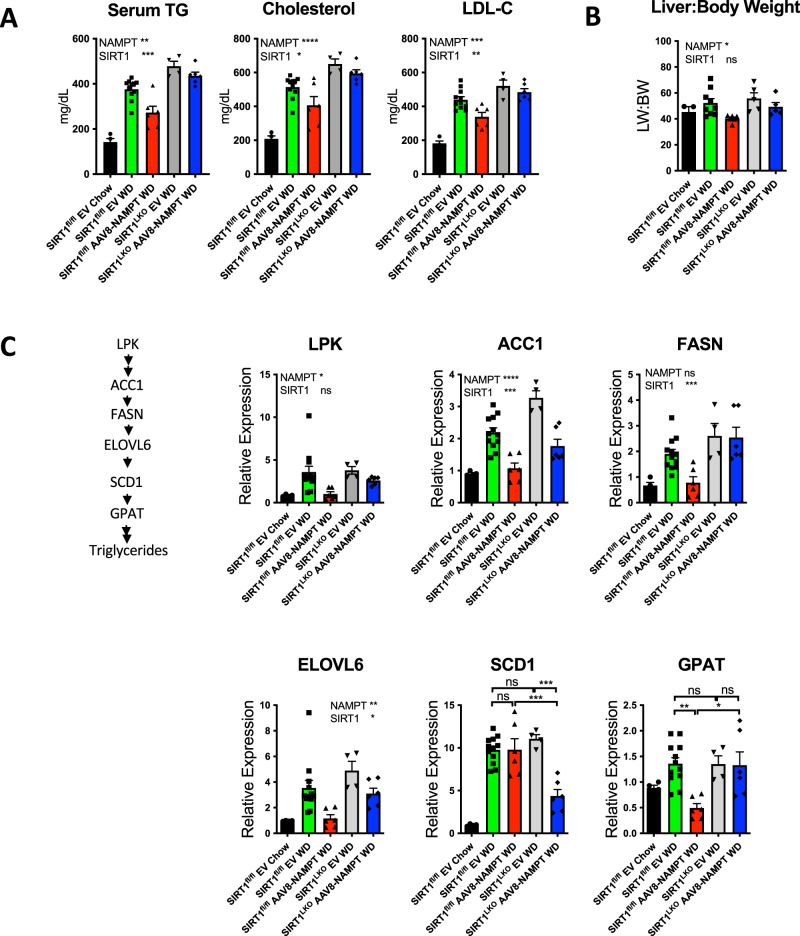
Hepatocyte SIRT1 selectively modulates anti-lipogenic effects of hepatocyte NAMPT overexpression. **A** Serum TG, cholesterol and LDL in Western diet-fed WT and SIRT1^LKO^ mice overexpressing empty vector or NAMPT. TG, triglycerides. LDL-C, low-density lipoprotein cholesterol. Interactions, *P* = ns for all circulating lipid analyses. *n* = 4 SIRT1^fl/fl^ Chow; 11 SIRT1^fl/fl^ EV WD; 6 SIRT1^fl/fl^ AAV8-NAMPT WD; 4 SIRT1^LKO^ EV WD; 6 SIRT1^LKO^ AAV8-NAMPT WD. **B** Liver weight-to-body weight ratios in mice analyzed in (**A**). Interaction *P* = ns. *n* = 3 SIRT1^fl/fl^ Chow; 9 SIRT1^fl/fl^ EV WD; 6 SIRT1^fl/fl^ AAV8-NAMPT WD; 5 SIRT1^LKO^ EV WD; 5 SIRT1^LKO^ AAV8-NAMPT WD. **C** De novo lipogenic gene expression in mice analyzed in (**B**). Linear substrate pathway is shown at left for illustrative purposes. Interaction *P* = ns for LPK, ACC1, FASN, ELOVL6. Interaction *P* < 0.05 and <0.001 for GPAT and SCD1. *, **, ***, *P* < 0.05, <0.01, <0.001 vs. bracketed control by Tukey’s multiple comparisons testing. *n* = 4 SIRT1^fl/fl^ Chow; 11 SIRT1^fl/fl^ EV WD; 6 SIRT1^fl/fl^ AAV8-NAMPT WD; 4 SIRT1^LKO^ EV WD; 6 SIRT1^LKO^ AAV8-NAMPT WD. Error bars in (**A**–**C**) represent SEM. Circles, SIRT1^fl/fl^ Chow; Squares, SIRT1^fl/fl^ EV WD; Upward Triangles SIRT1^fl/fl^ AAV8-NAMPT WD; Downward Triangles, SIRT1^LKO^ EV WD; Diamonds SIRT1^LKO^ AAV8-NAMPT WD. Statistical tests: (**A**–**C**), two-way ANOVA; **C** Tukey’s post hoc correction.

To better define NAMPT-SIRT1 signaling in lipid homeostasis in vivo, we examined hepatic and peripheral effects of hepatocyte NAMPT expression in WD-fed WT and SIRT1^LKO^ mice. NAMPT overexpression did not reduce serum transaminases or intrahepatic TG content in Western diet-fed mice (Supplementary Fig. 5A and B). Blinded histopathological analysis of livers in all Western diet-fed groups demonstrated simple steatosis without significant differences in NAS scoring or any of its component scores (e.g., steatosis, lobular inflammation, or ballooning, Supplementary Fig. 5C, D). In contrast, NAMPT and SIRT1 exerted independent effects on diet-induced dyslipidemia. Main effect analysis confirmed that hepatocyte NAMPT expression significantly reduced circulating TG and cholesterol (Fig. 7A), and liver weight-to-body weight ratio (Fig. 7B), whereas SIRT1 deficiency exacerbated the dyslipidemic effect of WD feeding without altering liver weight-to-body weight ratio. However, two-way analysis revealed that NAMPT and SIRT1 did not significantly interact to produce these effects (Fig. 7A, B). In addition, we observed differential SIRT1 and NAMPT interactions in regulating de novo lipogenic pathway gene expression. Main effect analysis here indicated that NAMPT expression reduced LPK, ACC1, and ELOVL6 expression, whereas SIRT1 deficiency independently exacerbated diet-induced ACC1, FASN and ELOVL6 expression (Fig. 7C). Conversely, NAMPT and SIRT1 significantly interacted to modulate GPAT expression as defined by two-way NAMPT-SIRT1 interaction. Post hoc analysis indicated significantly lower hepatocyte GPAT expression in NAMPT-expressing WT mice, whereas this effect was abrogated in SIRT1^LKO^ liver (Fig. 7C). Together, the data suggest that a NAMPT-SIRT1 axis mediates hepatocyte and circulating FGF21. Yet, surprisingly, the regulatory actions of NAMPT and SIRT1 in modulating hepatic and peripheral lipid homeostasis, largely occur in parallel.

### Transcriptomic analyses reveal tandem and parallel metabolic sequelae of hepatocyte NAMPT-SIRT1 interactions

**Fig. 8:**
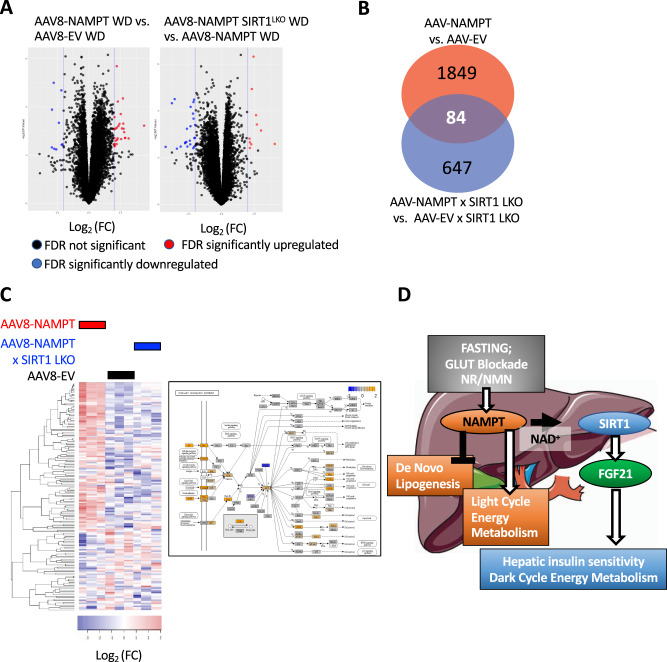
Hepatocyte NAMPT exerts SIRT1-dependent and SIRT-independent transcriptional effects. **A** Volcano plots showing differentially expressed genes from (threshold log(FC) = 2, FDR < 0.05) livers from WD-fed SIRT1^fll/fl^ NAMPT-overexpressing mice and WD-fed SIRT1^fll/fl^ vector controls (at left). Right, volcano plot showing differentially-expressed genes in livers from WD-fed NAMPT-overexpressing SIRT1^LKO^ mice vs. NAMPT-overexpressing SIRT1^fll/fl^ mice. *n* = 3 WD-fed SIRT1^fll/fl^ EV; 3 WD-fed SIRT1^fll/fl^ NAMPT; 3 WD-fed SIRT1^LKO^ AAV8-NAMPT. **B** Gene count of differentially expressed genes in livers from WD-fed SIRT1^fll/fl^ AAV-NAMPT vs. WD-fed SIRT1^fll/fl^ vector control mice (Red); differentially expressed genes in WD-fed SIRT1^LKO^ mice and WD-fed SIRT1^fll/fl^ mice overexpressing NAMPT (Blue). Common gene count is shown in their intersection (purple). *n* = 3 WD-fed SIRT1^fll/fl^ EV; 3 WD-fed SIRT1^fll/fl^ NAMPT; 3 WD-fed SIRT1^LKO^ AAV8-NAMPT. **C** Left, Unsupervised clustering of differentially expressed genes (Western diet-fed AAV8-NAMPT vs. Western diet-fed AAV8-NAMPT × SIRT1^LKO^). Right, pathway-based gene expression heatmap of the PI3K/AKT signaling pathway, a significantly upregulated GO pathway and CompBio NAMPT-SIRT1-dependent theme. Log_2_(FC) values are Western diet-fed AAV8-NAMPT vs. Western diet-fed AAV8-EV. *n* = 3 WD-fed SIRT1^fll/fl^ EV; 3 WD-fed SIRT1^fll/fl^ NAMPT; 3 WD-fed SIRT1^LKO^ AAV8-NAMPT. Heatmap color scales represent Log(FC). **D** Working model of hepatocyte NAMPT signaling and function. NAMPT nicotinamide phosphoribosyltransferase, SIRT1 sirtuin 1, FGF21 fibroblast growth factor 21, GLUT glucose transporter. Statistical tests: (**A**, **B**, **D**) EdgeR Exact, Benjamini–Hochberg post hoc correction.

Physiological data reveal complex serial and parallel metabolic signaling actions of hepatocyte NAMPT and SIRT1. Therefore, we sought to verify or refute our prior analyses by unbiased methods using transcriptomic liver analyses to further interrogate this selective interaction between hepatocyte NAMPT and SIRT1. Based on both molecular physiological and genetic data, we hypothesized that hepatocyte NAMPT exerts SIRT1-dependent and -independent transcriptional regulation. To that end, we subjected livers from WD-fed mice with or without NAMPT overexpression, and with or without hepatocyte-specific SIRT1 deficiency to transcriptomic analysis. The data demonstrated multiple significantly altered genes (FDR 0.05; Log (FC) > 2) in NAMPT-overexpressing mice when compared with vector-expressing control mice (Fig. 8A). The preponderance of these genes were upregulated, whereas hepatocyte SIRT1 deficiency yielded a majority of down-regulated genes when comparing the effect of NAMPT overexpression on WD-fed WT vs. SIRT1^LKO^ liver transcriptomes (Fig. 8A, left vs. right plots). This suggested that NAMPT conveys a transcriptionally stimulatory effect that depends, in part, on hepatocyte SIRT1. To define the broader NAMPT transcriptional regulation in the presence or absence of SIRT1, we quantified genes that are significantly altered (unadjusted *P* < 0.05) upon NAMPT-overexpression in WD-fed SIRT1^LKO^ mice and in WT mice. NAMPT overexpression in WD-fed WT mice significantly altered 1849 genes vs. vector controls, whereas NAMPT-overexpression in SIRT1^LKO^ mice increased 647 genes when compared with vector control SIRT1^LKO^ mice. 84 of these significantly altered transcript identities overlapped (Fig. 8B), delineating the presence of NAMPT-regulated transcripts that are both dependent and independent of SIRT1. To categorize these SIRT1-dependent and -independent transcriptomic alterations, we turned to CompBio (PercayAI, St. Louis, MO) in silico analysis. This algorithm searches significantly altered genes across peer-reviewed literature databases. It then groups genes into biological processes and pathways based on common recurrently associated concepts (Table 1). Agnostic in silico analysis revealed NAMPT-induced, SIRT1-dependent concepts that include hepatic PI3K/AKT signaling, NF- κB signaling, and regulation of T-cell signaling. SIRT1-dependent, NAMPT-suppressed included NASH-related and lipid and cholesterol and bile-acid metabolism-related transcript categories. In contrast, SIRT1-independent, NAMPT-upregulated processes included neutrophil chemotaxis and complement pathway concepts, whereas NAMPT suppressed de novo lipogenesis, hepatic steatosis, adipogenesis and retinoic acid pathways independent of SIRT1. A more granular view of the PI3K/AKT pathway as an exemplar NAMPT-SIRT1-dependent upregulated pathway revealed enhanced PI3K/AKT expression with their downstream targets in the FOXO and CREB pathways, without changes to the pro-lipogenic mTOR pathway (Fig. 8C). Overall, in silico transcriptomic analyses corroborate molecular physiological data, demonstrating that NAMPT exerts metabolic actions that occur both dependent upon and in parallel with its canonical hepatocyte protein deacetylase, SIRT1.

**Table 1:** CompBio in silico analysis of bulk RNA sequencing data showing significantly altered pathways in livers from NAMPT OE and WT controls.

	SIRT-dependent	SIRT1-independent
Increased NAMPT OE vs. WT	PI3K/AKT signaling NF*к*B Signaling T cell Regulation	Neutrophil Chemotaxis Complement Pathway
Decreased NAMPT OE vs. WT	NASH Lipid metabolism	De novo lipogenesis Hepatic Steatosis Retinoic acid Adipogenesis

## Discussion

Nutrient withdrawal creates an adapt-or-perish proposition for an organism in general, and for the hepatocyte in particular. The lack of exogenous glucose forces a reliance upon peripheral lipolysis and fatty acid oxidation in the hepatocyte. There are three issues that arise in the hepatocyte that must then be addressed at this point. First, electrons generated through the Krebs cycle must be shuttled into the electron transport chain, and this requires an electron carrier. Second, fasting induces a redox state imbalance, which necessitates redox equivalent generation. Third, the cell must, at least ideally, coordinate the changes in fuel reliance and utilization with mitochondrial biogenesis and fat oxidation. NAD^+^ biosynthesis and salvage unify the adaptive responses to each of these stresses in the substrate-restricted hepatocyte. Accordingly, NAD^+^ is a key redox and signaling intermediary that protects against diseases ranging from aging, and neurodegeneration to diabetes and non-alcoholic fatty liver disease (NAFLD)^25,57^. Here, we employ comprehensive mouse genetic and molecular physiologic approaches to dissect the necessity, sufficiency, and downstream mechanisms by which hepatocyte NAMPT modulates hepatic and extrahepatic metabolic homeostasis (Fig. 8D). We demonstrate that hepatocyte NAMPT is an important fasting-induced hepatic factor that exerts broad, adaptive transcriptional and metabolic effects on glucose-, energy-, and lipid homeostasis. Strikingly these effects both depend upon and occur in parallel to activation SIRT1.

The therapeutic effects of the hepatocyte SIRT1 pathway are generally established, and are rooted in several sets of in vivo data. First, hepatocyte SIRT1 overexpression improves hepatic fat oxidation and FGF21 secretion, adipose browning, and insulin sensitivity^24,28,32,58^. Concordantly, hepatocyte SIRT1 deficiency causes hepatic steatosis, insulin resistance and decreased energy expenditure^22,26–29^. In light of intense study surrounding hepatocyte functions and downstream metabolic actions of SIRT1, the upstream in vivo mechanisms of hepatocyte SIRT1 activation remained remarkably underappreciated. In the current study, we demonstrate that hepatocyte NAMPT activates hepatocyte SIRT1. In the absence of metabolic perturbations, NAMPT actions in healthy, young mice are modest in consequence (Supplementary Fig. 3). Yet, under duress of overnutrition, obesity and insulin resistance, hepatocyte NAMPT *selectively* activates SIRT1-dependent transcriptional regulation to attenuate diet-induced hepatic and extrahepatic metabolic derangements in energy homeostasis and glucose homeostasis.

This surprising result gives rise to at least three important implications. First, there are qualitative elements of SIRT1 activation which determine its downstream metabolic actions. Specifically, it seems to matter not only that SIRT1 is activated, but it also matters by what upstream mechanism(s) SIRT1 is activated. This is apparent when contrasting the broader SIRT1-driven effects of both intermittent fasting and hepatocyte SIRT1 overexpression^24,28,38,39,58^ with the targeted profile of SIRT1-dependent NAMPT effects that we demonstrate in the current work (Figs. 6, 7, and 8).

Second, we establish that hepatocyte NAMPT exerts adaptive metabolic effects apart from canonical SIRT1 activation. This includes NAMPT effects on aspects of peripheral glucose and energy homeostasis (Fig. 6), and hepatocyte and circulating lipid metabolism (Figs. 7 and 8), although subsequent work will more specifically elucidate NAMPT-SIRT1 interactions in glucose homeostasis. Nevertheless, the impact of these data is that they support deeper interrogation into how NAMPT modulates metabolism without its primary NAD^+^-dependent effector. One observation upon which we can begin to hypothesize such mechanisms is that hepatocyte SIRT1 deficiency diminishes—but does not completely abrogate—hepatic or circulating FGF21 (Fig. 6E). The applicable hypothesis is, therefore, that NAMPT induces SIRT1-independent FGF21, and this remnant activation is sufficient to drive thermogenic, glucose, and lipid metabolic effects apart from direct SIRT1 regulation. An arguably more intriguing alternate hypothesis is that hepatocyte and/or extracellular NAMPT mediate additional NMN- or NAD^+^-dependent processes (e.g., SIRT1, or other sirtuin family member activation) in extrahepatic tissues^25,45,46,59^, to mediate parallel metabolic sequelae.

Finally, the data indicate that inducing the SIRT1 pathway at or proximal to the level of NAMPT may optimally leverage the broader effects of NAMPT-SIRT1 signaling, and other fasting-like intermediaries. For example, restricting hepatocyte glucose entry also conveys much of the adaptive generalized fasting response, which includes NAMPT-SIRT1 activation^5–12,60^. Here, and in our prior work, we demonstrated that both acute fasting and blocking hepatic glucose transport in vitro and in vivo activate hepatocyte NAMPT and adipose browning (Fig. 1 and Ref. ^50^), and also reduce hepatic steatosis^5–7,11,13,15,53^. These hepatocyte-centered approaches could complement broader NAMPT-SIRT activation approaches via NAD^+^ precursor administration^44,49,59,61–63^ to best approximate and augment fasting-like metabolic effects.

It is also important to note here that these data shed light on both acute and prolonged fasting (e.g., torpor). The preponderance of reported data here are from 0 to 24 h fasting mice (Fig. 2), which contextualizes the data with important foundational studies that precede this work^28,45,64^. In addition, new data from prolonged fasting mice (e.g., up to 48 h fasting, Fig. 2A, B) indicate that hepatocyte NAMPT regulates fasting thermogenesis and fuel utilization during both acute and prolonged fasting states^28,45,64^.

Overall, our data demonstrate that NAMPT is necessary and sufficient to enhance thermal, lipid and glucose homeostasis upon metabolic duress. We provide evidence that hepatocyte NAMPT activates hepatocyte SIRT1 and FGF21 to specify and refine the ensuing metabolic program, yet remarkably, SIRT1 activation is only selectively leveraged downstream of NAMPT. These data highlight a direct therapeutic role for hepatocyte NAMPT activation downstream of generalized fasting or hepatocyte GLUT inhibition, which can be utilized against obesity, aging, neurodegenerative, and other fasting-labile diseases.

## Methods

### Animal studies

All animal protocols were approved by the Washington University School of Medicine Animal Studies Committee. Male C57B/6J mice (#00664), *Sirt1*^LoxP/LoxP^ (#029603^65^), Albumin-cre (#003574) mice, and *db/db* (#003574) mice were purchased directly from the Jackson Laboratory (Bar Harbor, ME) and housed a 12 h alternating light-dark, temperature-controlled, specific pathogen-free barrier facility prior to and throughout experimentation. We obtained permission to study the *Nampt*^LoxP/LoxP^ mice as a gift from Dr. Oberdan Leo (Université Libre de Bruxelles^66^), and the mice were physically provided to us from Dr. Shin Imai’s laboratory (Washington University). Albumin-cre mice were bred with *Sirt1*^*LoxP/LoxP*^, and *Nampt*^*LoxP/LoxP*^ mice to generate germ-line, hepatocyte-specific target excision (SIRT1^LKO^ and NAMPT^LKO^ mice). Our breeding scheme for all LKO experiments was *Sirt1*^*LoxP/LoxP*^ × *Sirt1*^*LoxP/LoxP*^/Alb*Cre*^+/wt^, and *Nampt*^*LoxP/LoxP*^ × *Nampt*^LoxP/LoxP^/Alb*Cre*^+/wt^. This produced a ~1:1 ratio of mice wild-type and knockout at each locus specifically in hepatocytes. Experimental mice of each genotype were co-housed throughout dietary intervention to minimize cage-specific and microbiotic effects.

Procedures were performed in accordance with approved guidelines by the Animal Studies Committee (Washington University School of Medicine). Animal studies were performed in accordance with ethical regulations outlined by the Institutional Animal Care and Use Committee (IACUC).

### NAD^+^ quantification

NAD^+^ was extracted from frozen mouse tissue samples using cold perchloric acid. NAD^+^ concentrations were determined using an HPLC system (Prominence; Shimadzu Scientific Instruments, Columbia, MD) with a Supelco LC-18-T column (#58970-U; Sigma, St. Louis, MO) as described previously^42,45^. NAD^+^ concentrations were normalized to input tissue weight.

### Dietary treatment

Mice were fed with standard rodent chow or western diet (Teklad # TD88137) ad libitum for up to 12 weeks. During the entire treatment course, mice were given free access to sterilized water.

### AAV8- and adenovirus-mediated overexpression

AAV8 and adenovirus were administered via tail vein as we previously reported. 10^9^ particles per dose (adenovirus) and 10^11^ particles (AAV8) were delivered, each dissolved in 150 uL total injection volume per animal as we reported^8,9,50,51^. We optimized expression conditions for assay of adenovirus- and AAV8-treated mice 48h- and 10d post treatment, respectively. We also optimized conditions to achieve 1.5-2.0-fold hepatocyte NAMPT overexpression, which aligns with physiologic NAMPT upregulation during 12 h fasting or after 5d oral trehalose treatment (Supplementary Fig. 6) All viral vectors were obtained directly from Vector Biolabs Inc. (Philadelphia, PA).

### Primary hepatocyte isolation, culture and treatment

Primary murine hepatocytes obtained from WT mice were isolated^24,25,31^ and cultured and maintained in regular DMEM growth media (Sigma, #D5796) containing 10% FBS. For in vitro starvation experiments, starve media contained 1 g/L glucose and 0.5% FBS was used. For trehalose treatment assays, trehalose stimulation was 100 mM in Fig. 1D and 10 mM and 100 mM in Fig. 1F. These are physiologically relevant concentrations relative to portal venous trehalose concentrations, as we recently reported in Zhang et al., *Gastroenterology* 2020. Total culture time was 36 h for the trehalose in vitro experiments. Briefly, we plated 1 * 10^6^ cells per well in six-well plates, which yielded 70–80% confluent plates for experimentation. 12 h after plating, cells were treated with fructose in the presence or absence of trehalose or LT for 24 h. For the in vitro experiments in Supplemental Fig. 2 we seeded six-well plates containing 1 * 10^6^ cells per well (70–80% confluent plates). 12 h after plating, cells were transfected and assayed 48 h later (e.g., 60 h total plating time). All cultures were lysed in Trizol and subjected to downstream analysis.

### Ex vivo adipose tissue treatment

Sub-cutaneous adipose tissue was dissected from 6 to 8wk-old WT mice and incubated 24 h in regular growth media (DMEM + 4.5 g/L glucose + 10% FCS) with vehicle (0.1% BSA) or with 100 ng/mL recombinant murine FGF21 (R&D Systems, Minneapolis, MN). Explants were washed in ice-cold PBS and snap-frozen in PBS prior to analysis.

### Quantitative real-time RT-PCR (qRT-PCR)

Total RNA was prepared by homogenizing snap-frozen livers or cultured hepatocytes in Trizol reagent (Invitrogen #15596026) according to the manufacturer’s protocol. cDNA was prepared using Qiagen Quantitect reverse transcriptase kit (Qiagen #205310). Real-time qPCR was performed with Step-One Plus Real-Time PCR System (Applied Biosystems) using SYBR Green master Mix Reagent (Applied Biosystems) and specific primer pairs. Relative gene expression was calculated by a comparative method using values normalized to the expression of an internal control gene. Primer sequences are in Supplementary Table 1^54^.

### Immunoblotting and ELISA

Immunoblotting and FGF21 and Insulin ELISAs were performed precisely as described^10,11,50^. Protein expression levels were quantified with Image Lab software and normalized to the levels of β-actin, transferrin, or GAPDH.

### Antibodies

Antibodies against GAPDH (#5174), SIRT1 (#2028), vinculin E1E9V (#13901), and acetyl-lysine (#9441) were purchased from Cell Signaling Technology (CST, Beverly, MA, USA). NAMPT (#ab236874), UCP1 (#ab155117) and FGF21 (#ab171941) antibodies were obtained from Abcam (Cambridge, MA). The dilution for all primary antibodies was 1:1,000 in 5% non-fat milk in tris-buffered saline with tween 20 (TBST). Secondary antibodies were peroxidase-conjugated anti-rabbit and anti-mouse IgG (CST, 1:5,000-1:10,000 dilution).

### Histological analysis

Formalin-fixed paraffin-embedded liver sections were stained by H&E via the Washington University Digestive Diseases Research Core Center as we reported previously^9–11,51^. A treatment-blinded, board-certified GI/Liver pathologist scored each liver section for inflammation, steatosis, and ballooning^67^.

### Insulin and glucose tolerance testing

For insulin tolerance tests (ITT), mice were injected with 0.75 IU per kg body weight of insulin (Humalog, Eli Lilly) intraperitoneally after 4 h of fasting on Aspen bedding. For glucose tolerance tests (GTT), mice were injected with 2 g per kg body weight of glucose intraperitoneally after fasting for 4 h on aspen bedding. *db/db* mice were injected with 1 g per kg body weight of glucose intraperitoneally after fasting for 16 h on aspen bedding. Blood samples were measured at different time points with a glucometer (Arkray USA, Inc., Minneapolis, MN, USA).

### Clinical chemistry and hepatic lipid analyses

For serum analyses, submandibular blood collection was performed immediately prior to sacrifice and serum was separated. Triglycerides (Thermo Fisher Scientific #TR22421) and Cholesterol (Thermo Fisher Scientific #TR13421) quantification were performed using commercially available reagents according to manufacturer’s directions.

Hepatic lipids were extracted from ~100 mg hepatic tissue homogenized in 2:1 chloroform:methanol. 0.25–0.5% of each extract was evaporated overnight prior to biochemical quantification of triglycerides, LDL-C, cholesterol, and free fatty acids using reagents described above, precisely according to manufacturer’s directions.

### Body composition analysis

Body composition analysis was carried out in unanesthetized mice using an EchoMRI 3-1 device (Echo Medical Systems, Houston, TX) via the Washington University Diabetic Mouse Models Phenotyping Core Facility.

### Indirect calorimetry and food intake measurement

All measurements were performed in a PhenoMaster System (TSE systems) via the Washington University Diabetic Mouse Models Phenotyping Core Facility, which allowed metabolic performance measurement and activity monitoring by an infrared light-beam frame. Mice were placed at room temperature (22–24 °C) in separate chambers of the PhenoMaster open-circuit calorimetry. Mice were allowed to acclimatize in the chambers for 24 h. Food and water were provided ad libitum in the appropriate devices. The parameters of indirect calorimetry (VO_2_, VCO_2_, RER), heat and movement) were measured for at least 24 h for a minimum of one light cycle (06:01–18:00) and one dark cycle (18:01–06:00). Presented data are average values obtained in these recordings.

### Transcriptomic analysis

RNA sequencing was performed by the Washington University Genome Technology Access Center) as we reported^51^. Library preparation was performed with 10 μG of total RNA with a Bioanalyzer RIN score >8.0. Ribosomal RNA was removed by poly-A selection using Oligo-dT beads (mRNA Direct kit, Life Technologies). mRNA was fragmented and reverse transcribed to yield cDNA using SuperScript III RT enzyme (Life Technologies) and random hexamers. A second strand reaction was performed to yield ds-cDNA, and then had Illumina sequencing adapters ligated to the ends. Ligated fragments were amplified for 12 cycles and sequenced on an Illumina HiSeq-3000 using single reads extending 50 bases. RNA-seq reads were aligned to the Ensembl release 76 top-level assembly with STAR version 2.0.4b. Gene counts were derived from the number of uniquely aligned unambiguous reads by Subread:featureCount version 1.4.5. Transcript counts were produced by Sailfish version 0.6.3.

### Tool description

The PercayAI Software Platform performs a literature analysis to identify relevant biological processes and pathways represented by the differentially expressed entities (genes, proteins, miRNAs, or metabolites). The PercayAI Software extracts all abstracts from PubMed that reference entities of interest (or their synonyms), using contextual language processing and a biological language dictionary that is not restricted to fixed pathway and ontology knowledge bases. Conditional probability analysis is utilized to compute the statistical enrichment of biological concepts (processes/pathways) over those that occur by random sampling. Related concepts built from the list of differentially expressed entities are further clustered into higher-level themes (e.g., biological pathways/processes, cell types and structures, etc.).

### Scoring description

Within the PercayAI Software Platform, scoring of gene, concept, and overall theme enrichment is accomplished using a multi-component function referred to as the Normalized Enrichment Score (NES). The first component utilizes an empirical p-value derived from several thousand random entity lists of comparable size to the users input entity list to define the rarity of a given entity-concept event. The second component, effectively representing the fold enrichment, is based on the ratio of the concept enrichment score to the mean of that concept’s enrichment score across the set of randomized entity data.

The input criteria here were as follows: Biological processes and pathways identified from genes that were both upregulated upon NAMPT overexpression and downregulated in NAMPT-overexpressing SIRT1^LKO^ mice were labeled as SIRT1-Dependent. Biological processes and pathways identified from genes that were upregulated in NAMPT-overexpressing WT, but not downregulated in NAMPT-overexpressing SIRT1^LKO^ mice were labeled as SIRT1-Independent. Conversely, biological processes and pathways that were both downregulated upon NAMPT overexpression and upregulated in NAMPT-overexpressing SIRT1^LKO^ mice were labeled as SIRT1-Dependent. Biological processes and pathways that were down-regulated upon NAMPT overexpression in both WT and SIRT1^LKO^ liver were again labeled as SIRT1-independent.

### Statistical analyses

Data were analyzed using GraphPad Prism version 9.0 *p* < 0.05 was defined as statistically significant. Data shown are as mean ± SEM. In dot plots: the horizontal line represents the data mean. Thermogenesis data were analyzed by analysis of covariance (ANCOVA) using body weight as the covariate. Two-tailed homoscedastic *T*-testing with Bonferroni post hoc correction for multiple comparisons was used for paired analyses. Two-way ANOVA was used for analysis of statistical interactions between two independent variables (e.g., NAMPT/SIRT1). Interaction *P* values are reported in corresponding Figure Legends. Significant two-way interactions prompted Tukey’s multiple comparison analyses to compare specific group means. These post hoc results are placed within each panel, with brackets denoting specific comparisons. In cases wherein no significant two-way interaction is detected, only main effects are reported, and these are demonstrated within each panel.

Studies requiring repeated sampling from the same animal over time (e.g., body weight vs. time, glucose- and insulin tolerance testing), data were analyzed as repeated-measure mixed models with factor, time, and factor*time interaction as fixed effects. Subject within factor was designated as a random effect. Correlation of repeated measurements within subject were accounted for with a first-order autocorrelation covariance structure.

For RNAseq analyses, gene counts were imported into the R/Bioconductor package EdgeR and TMM normalization size factors were calculated to adjust for samples for differences in library size. Ribosomal genes and genes not expressed in the smallest group size minus one samples greater than one count-per-million were excluded from further analysis. The TMM size factors and the matrix of counts were then imported into the R/Bioconductor package Limma. Weighted likelihoods based on the observed mean-variance relationship of every gene and sample were then calculated for all samples by voomWithQualityWeights. The performance of all genes was assessed with plots of the residual standard deviation of every gene to their average log-count with a robustly fitted trend line of the residuals. Differential expression analysis was then performed to analyze for differences between conditions and the results were filtered for only those genes with Benjamini–Hochberg false-discovery rate adjusted *p* values ≤ 0.05.

### Reporting summary

Further information on research design is available in the Nature Research Reporting Summary linked to this article.

## Supplementary information

Supplementary Information

Reporting Summary

## Data Availability

There are no restrictions on data or material availability. Data are available upon reasonable request. The RNAseq data in Fig. 2 used in this study are available in the GEO database under accession code GSE184395. AdNAMPT db/db data generated in this study in Fig. 4 have been deposited in the GEO database under accession code GSE184513. The RNAseq data generated in this study and shown in Fig. 8 have been deposited in the GEO database under accession code GSE184394. All other data generated or analyzed during this study are included in this published article (and its supplementary information files). Source data are provided with this paper.

## Source data

Source Data
