# Supplementary material for: SIRT1 selectively exerts the metabolic protective effects of hepatocyte nicotinamide phosphoribosyltransferase
Source: Nat Commun. 2022 Feb 28;13:1074. doi: 10.1038/s41467-022-28717-7 (PMC8885655; doi:10.1038/s41467-022-28717-7)
Supplement: Supplementary file 1 — Supplementary Information [file 41467_2022_28717_MOESM1_ESM.pdf]

Supplemental Fig. 1

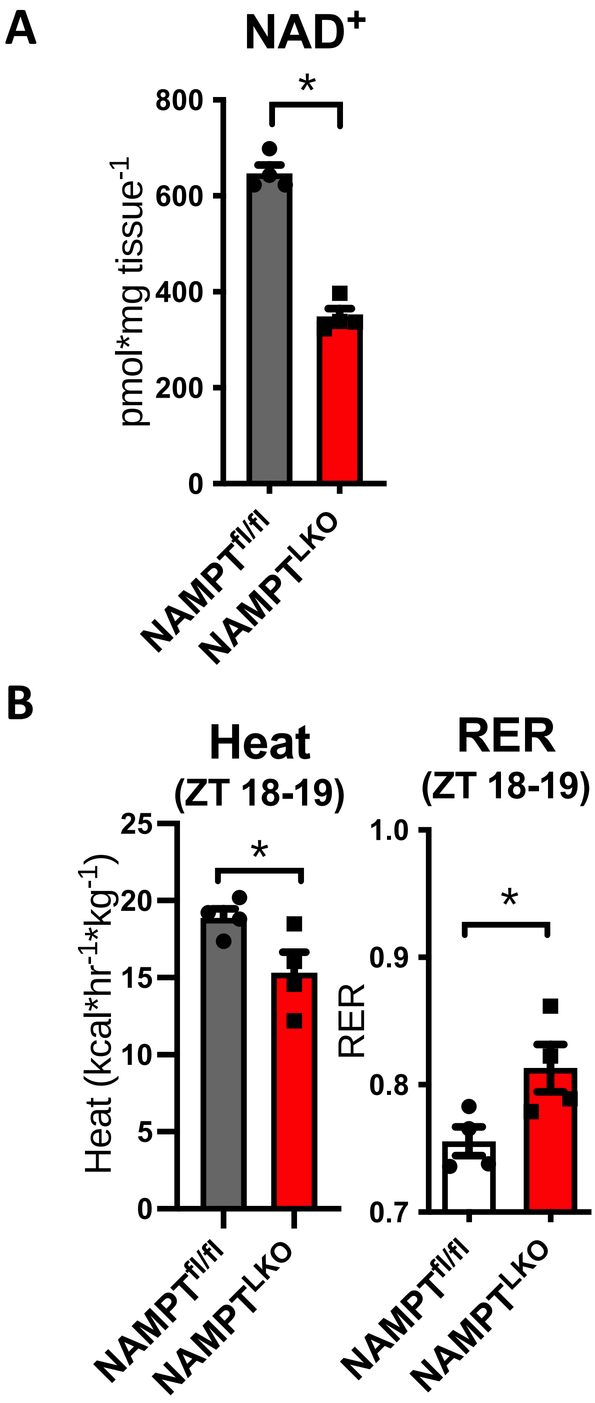

Supplementary Figure 1. Hepatocyte NAMPT mediates [NAD$^+$] accumulation and fasting heat generation. A. NAD$^+$ quantification by high-performance liquid chromatography in livers from random-fed NAMPT$^{fl/fl}$ or NAMPT^{LKO} mice. n = 4 NAMPT$^{fl/fl}$; 4 NAMPT^{LKO} mice. B. Analysis of mean 1h heat generation and RER by indirect calorimetry obtained during ZT 18-19 of the first fasting period in WT and hepatocyte-specific NAMPT deletion mutant (NAMPT^{LKO}) mice. The data represent mean heat and RER after 6h fasting. *, $P < 0.05$ vs control. n = 4 NAMPT$^{fl/fl}$; 4 NAMPT^{LKO} mice. Error bars in A. and B. represent SEM. Circles, NAMPT$^{fl/fl}$, Squares, NAMPT^{LKO} mice.

Statistical tests: S1A, S1B, 2-tailed T-test.

Supplemental Fig. 2

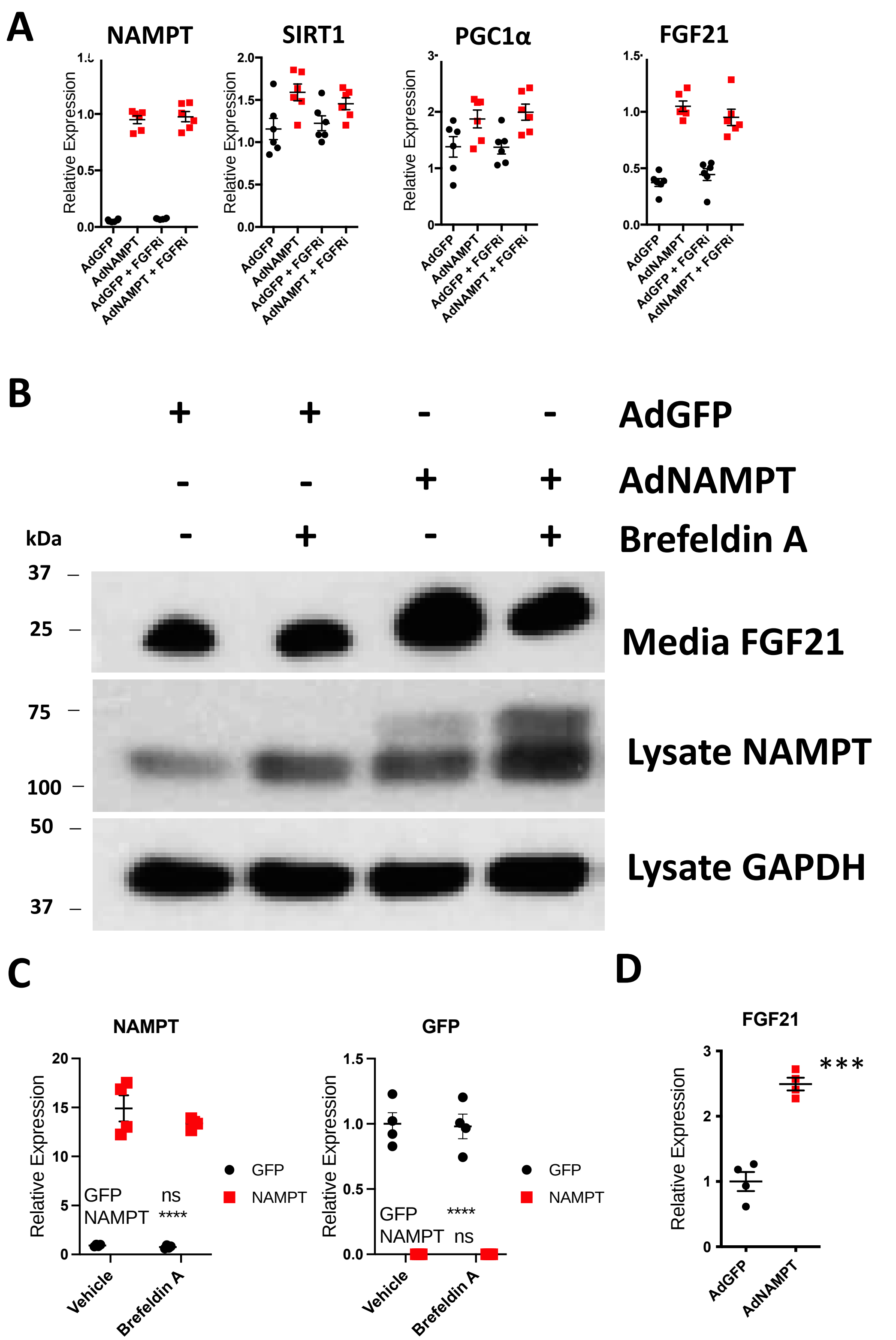

Supplementary Figure 2. NAMPT overexpression increases hepatocyte FGF21 mRNA and FGF21 release in isolated hepatocytes. A. Fasting-like gene expression in primary murine hepatocytes overexpressing GFP or NAMPT in the presence or absence of the FGFR inhibitor, LY2874455. n = 6 GFP; 6 NAMPT; 6 GFP/FGFRi; 6 NAMPT/FGFRi. B. FGF21 release and NAMPT accumulation in primary murine hepatocytes overexpressing GFP or NAMPT in the presence or absence of pretreatment with the exocytosis inhibitor brefeldin A. n = 4 GFP; 4 GFP/BrefA; 4 NAMPT; 4 NAMPT BrefA (Representative Blot from two independent experiments). C. NAMPT and GFP mRNA expression in primary murine hepatocytes treated with or without the exocytosis inhibitor, brefeldin A (BFA). n = 4 GFP; 4 GFP/BrefA; 4 NAMPT; 4 NAMPT/BrefA D. FGF21 mRNA in isolated hepatocytes expressing GFP or NAMPT. n = 4 AdGFP; 4 AdNAMPT. ****, P < 0.0001 vs. bracketed control. Error bars in A, C, and D. represent SEM. Circles, cultures overexpressing GFP, Squares, cultures overexpressing NAMPT. Statistical tests: S2A, S2C, S2D 2-tailed T-test.

Supplemental Fig. 3

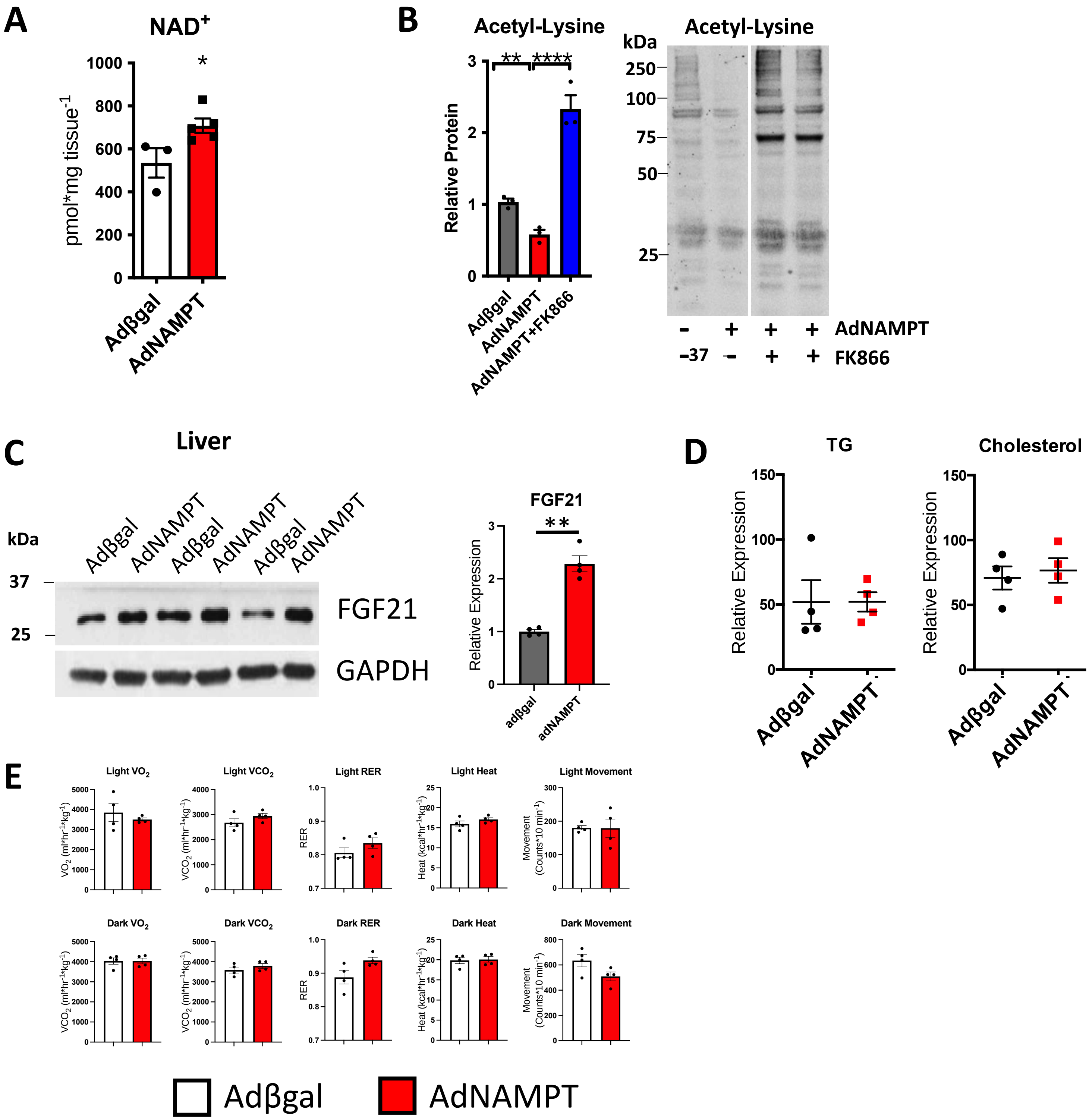

Supplementary Figure 3. Hepatocyte NAD+ links NAMPT induction with NAD+-dependent deacetylase activity and hepatic FGF21 expression. A. [NAD+] in livers of βGal (Circles) and NAMPT (squares)-overexpressing mice (48h). *, $P < 0.05$ by 2-tailed T-test. n = 3 AdβGal; 5 AdNAMPT. B. Representative acetyl-lysine immunoblot and densitometry in cultured AML12 hepatocytes transfected with β-gal or NAMPT prior to treatment with or without the NAMPT inhibitor, FK866 (100nM). n = 3 AdβGal; 3 AdNAMPT; 3 AdNAMPT/FK866. ** , **** $P < 0.01$ and < 0.0001 vs. AdNAMPT by one-way ANOVA and Dunnett's multiple comparisons test. C. Representative FGF21 immunoblot (left) and qPCR (right) quantification in livers from mice expressing β-galactosidase or NAMPT. Data from n = 4 AdβGal; 4 AdNAMPT mice. *, **, $P < 0.05$ and < 0.01 vs. control by 2-tailed T-test. D. Serum TG and cholesterol by enzymatic-colorimetric assay in chow-fed, WT mice treated with adenovirus encoding β-galactosidase (Circles) or NAMPT (Squares). n = 4 AdβGal; 4 AdNAMPT. E. Light and dark cycle VO_2, VCO_2, respiratory exchange ratio (RER), heat generation and movement in wild-type mice overexpressing β-gal or NAMPT. n = 4 AdβGal; 4 AdNAMPT. *$P < 0.05$, **$P < 0.01$, ****$P < 0.0001$.

Statistical tests: S3B, 1-way ANOVA, Dunnett's post hoc test. S3A, S3C, S3D, S3E 2-tailed T-test. Error bars in A-D represent SEM.

Supplemental Fig. 4

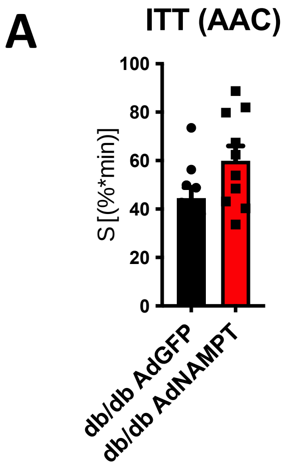

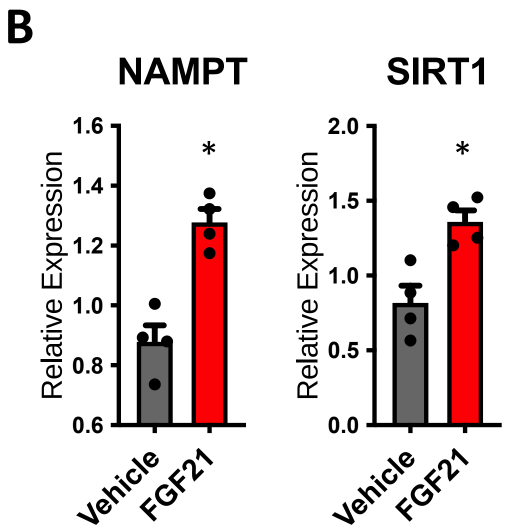

Supplementary Figure 4. A. ITT area-above-curve (AAC) calculation for insulin tolerance testing curves shown in Fig. 4K. n = 10 mice per group. Circles, db/db AdGFP mice, Squares, db/db AdNAMPT mice. B. FGF21 induces WAT NAMPT and SIRT1 expression. Subcutaneous WAT from WT mice was incubated with vehicle or recombinant murine FGF21 (24h, 100ng/mL). NAMPT and SIRT1 mRNA were quantified by qRT-PCR. *, $P < 0.05$ versus vehicle-treated. n = 4 vehicle; 4 FGF21-treated. Error bars in A and B represent SEM.

Statistical test: 2-tailed T-test.

Supplemental Fig. 5

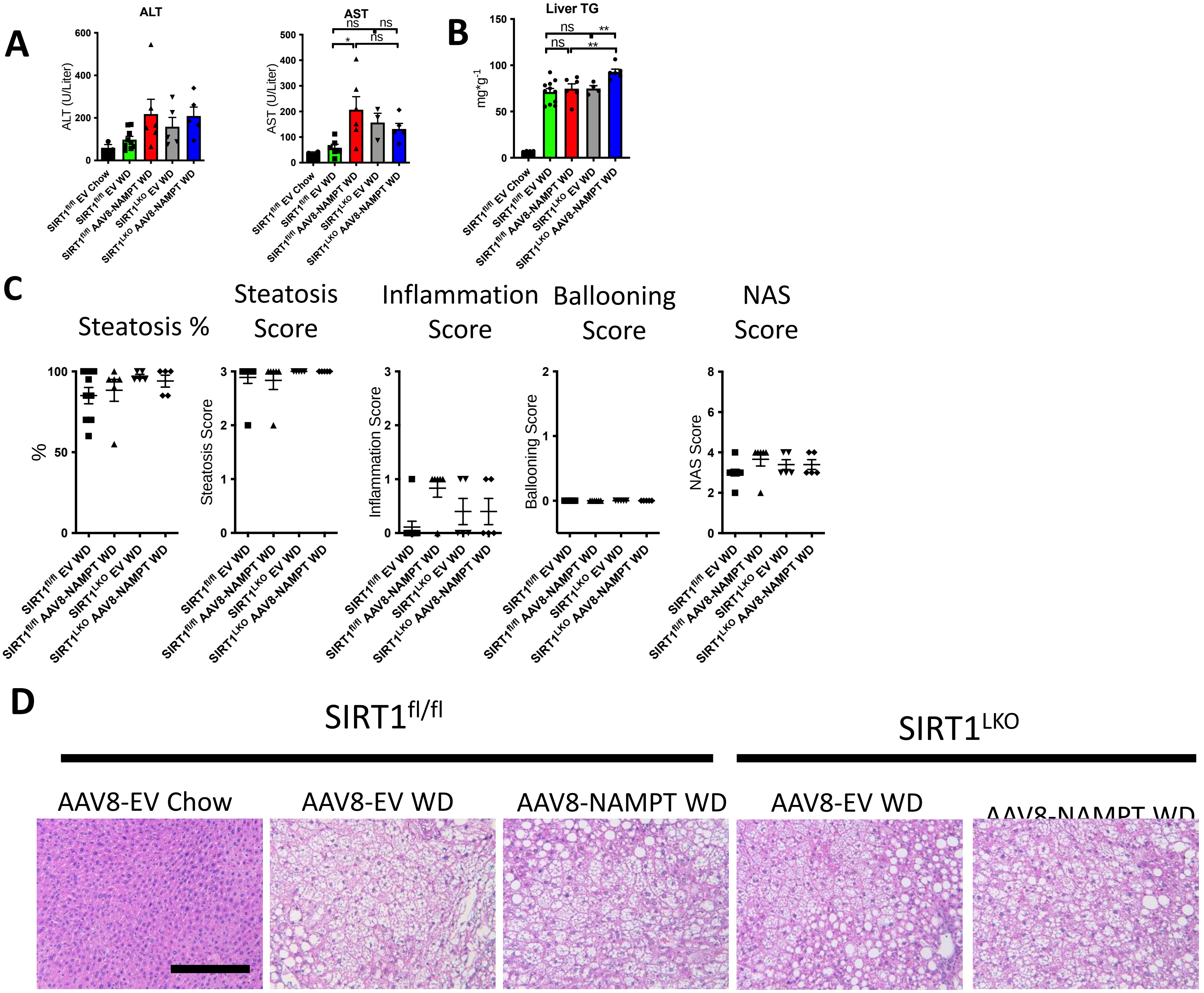

Supplementary Figure 5. No inflammatory infiltrate in chow- or WD-fed livers of WT and SIRT1^{LKO} mice overexpressing control vector (EV) or NAMPT. A. Serum ALT and AST in WD-fed WT and SIRT1^{LKO} mice overexpressing control vector or NAMPT. Interaction P = ns and < 0.05 for ALT and AST respectively. *, P < 0.05 versus bracketed control by Tukey's multiple comparison testing. ALT n = 3 SIRT1$^{fl/fl}$ Chow; 9 SIRT1$^{fl/fl}$ EV WD; 6 SIRT1$^{fl/fl}$ AAV8-NAMPT WD; 5 SIRT1^{LKO} EV WD; 5 SIRT1^{LKO} AAV8-NAMPT WD. AST n = 2 SIRT1$^{fl/fl}$ Chow; 6 SIRT1$^{fl/fl}$ EV WD; 6 SIRT1$^{fl/fl}$ AAV8-NAMPT WD; 3 SIRT1^{LKO} EV WD; 5 SIRT1^{LKO} AAV8-NAMPT WD. B. Liver TG in mice analyzed in (A). n = 4 SIRT1$^{fl/fl}$ Chow; 11 SIRT1$^{fl/fl}$ EV WD; 6 SIRT1$^{fl/fl}$ AAV8-NAMPT WD; 4 SIRT1^{LKO} EV WD; 6 SIRT1^{LKO} AAV8-NAMPT WD. C. Treatment-blinded histopathological scoring of liver sections from WD-fed WT and SIRT1^{LKO} mice overexpressing control vector or NAMPT. P = ns for all interactions shown. P = NS for NAMPT and SIRT as determinant factors for each Steatosis or Inflammation (scoring range 0-3), Ballooning (range 0-2) or NAS (range 0-8). n = 9 SIRT1$^{fl/fl}$ EV WD; 6 SIRT1$^{fl/fl}$ AAV8-NAMPT WD; 5 SIRT1^{LKO} EV WD; 5 SIRT1^{LKO} AAV8-NAMPT WD. D. High-powered micrographs of hematoxylin & eosin-stained liver sections from WD-fed WT and SIRT1^{LKO} mice overexpressing control vector or NAMPT. 2-way ANOVA interaction P < 0.05 for hepatic TG. Scale Bar, 200 μm. n = 4 SIRT1$^{fl/fl}$ Chow; 9 SIRT1$^{fl/fl}$ EV WD; 6 SIRT1$^{fl/fl}$ AAV8-NAMPT WD; 5 SIRT1^{LKO} EV WD; 5 SIRT1^{LKO} AAV8-NAMPT WD. Error bars in A-C represent SEM. Circles, SIRT1$^{fl/fl}$ Chow; Squares, SIRT1$^{fl/fl}$ EV WD; Upward Triangles SIRT1$^{fl/fl}$ AAV8-NAMPT WD; Downward Triangles, SIRT1^{LKO} EV WD; Diamonds SIRT1^{LKO} AAV8-NAMPT WD.

Statistical tests: S5A, S5B, S5C, 2-way ANOVA with Tukey's post hoc testing.

Supplemental Fig. 6

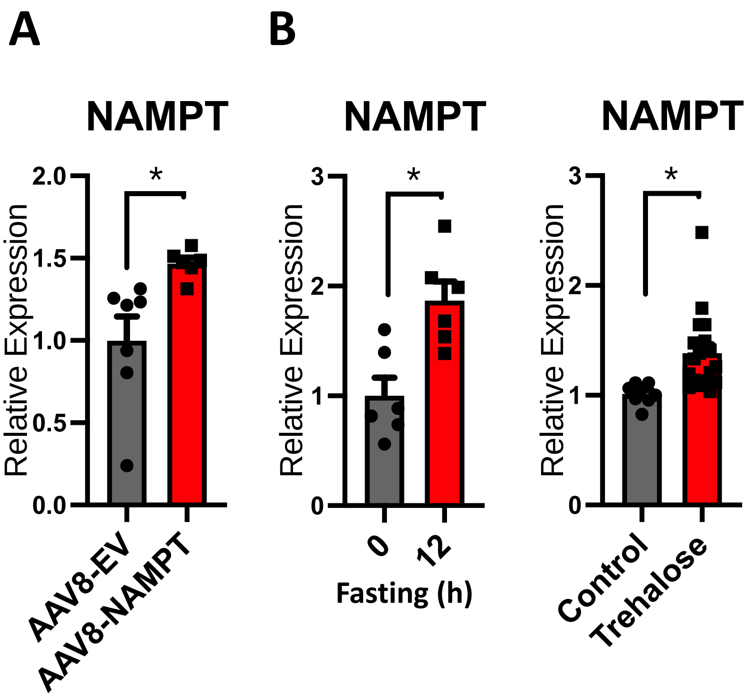

Supplementary Figure 6. AAV8-NAMPT optimization to reflect physiological NAMPT overexpression. A. NAMPT expression in livers of mice treated via tail vein with 10^{11} AAV8 particles encoding control vector (AAV8-EV, circles) or AAV8-NAMPT (squares) prior to liver qRT-PCR analysis. n = 7 AAV8-EV; 6 AAV8-NAMPT mice B. Comparison of hepatic NAMPT expression after 0h (n = 6) or 12h (n = 6) fasting (Circles and squares, respectively), or after 5d trehalose oral treatment. n = 8 vehicle (circles); 18 trehalose-treated mice (NAMPT). *, **, $P < 0.05$ and < 0.01 vs. control. Error bars in A and B represent SEM.

Statistical tests: S6A, S6B (both sub-panels), 2-tailed T-test.

Supplemental Table 1

cDNA Target	Forward (5' - 3')	Reverse (5' - 3')
Acc1	TGT CCG CAC TGA CTG TAA CCA	TGC TCC GCA CAG ATT CTT CA
Fgf21	CTG CTG GGG GTC TAC CAA G	CTG CGC CTA CCA CTG TTC C
Pgc1α	ACA CCG CAA TTC TCC CTT GT	CGG CGC TCT TCA ATT GCT TT
Ucp1	ACA CCG CAA TTC TCC CTT GT	CGG CGC TCT TCA ATT GCT TT
Nampt	GCA GAA GCC GAG TTC AAC ATC	TTT TCA CGG CAT TCA AAG TAG GA
Lpk	CTT GCT CTA CCG TGA GCC TC	ACC ACA ATC ACC AGA TCA CC
Fasn	CCT GGA TAG CAT TCC GAA CCT	AGC ACA TCT CGA AGG CTA CAC A
Elovl6	GAA AAG CAG TTC AAC GAG AAC G	AGA TGC CGA CCA CCA AAG ATA
Scd1	CCG GAG ACC CTT AGA TCG A	TAG CCT GTA AAA GAT TTC TGC AAA CC
Gpat	CAA CAC CAT CCC CGA CAT C	GTG ACC TTC GAT TAT GCG ATC A
Sirt1	GGG TGT CTG TTT CAT GTG GA	AAT CAG GCA AGA TGC TGT TG
Prdm16	AGC CCT CGC CCA CAA CTT GC	TGA CCC CCG GCT TCC GTT CA

Supplementary Table 1. Primers sequences used in this study.
