# Supplementary material for: SIRT1 selectively exerts the metabolic protective effects of hepatocyte nicotinamide phosphoribosyltransferase
Source: Nat Commun. 2022 Feb 28;13:1074. doi: 10.1038/s41467-022-28717-7 (PMC8885655; doi:10.1038/s41467-022-28717-7)
Supplement: Supplementary file 2 — Reporting Summary [file 41467_2022_28717_MOESM2_ESM.pdf]

nature research

Corresponding author(s): Brian J DeBosch, MD, PhD

Reporting Summary

Nature Research wishes to improve the reproducibility of the work that we publish. This form provides structure for consistency and transparency in reporting. For further information on Nature Research policies, see Authors & Referees and the Editorial Policy Checklist.

Statistical parameters

When statistical analyses are reported, confirm that the following items are present in the relevant location (e.g. figure legend, table legend, main text, or Methods section).

n/a	Confirmed	
☐	☒	The exact sample size (n) for each experimental group/condition, given as a discrete number and unit of measurement
☐	☒	An indication of whether measurements were taken from distinct samples or whether the same sample was measured repeatedly
☐	☒	The statistical test(s) used AND whether they are one- or two-sided<br>*Only common tests should be described solely by name; describe more complex techniques in the Methods section.*
☐	☒	A description of all covariates tested
☐	☒	A description of any assumptions or corrections, such as tests of normality and adjustment for multiple comparisons
☐	☒	A full description of the statistics including central tendency (e.g. means) or other basic estimates (e.g. regression coefficient) AND variation (e.g. standard deviation) or associated estimates of uncertainty (e.g. confidence intervals)
☐	☒	For null hypothesis testing, the test statistic (e.g. F, t, r) with confidence intervals, effect sizes, degrees of freedom and P value noted<br>*Give P values as exact values whenever suitable.*
☒	☐	For Bayesian analysis, information on the choice of priors and Markov chain Monte Carlo settings
☐	☒	For hierarchical and complex designs, identification of the appropriate level for tests and full reporting of outcomes
☒	☐	Estimates of effect sizes (e.g. Cohen's d, Pearson's r), indicating how they were calculated
☐	☒	Clearly defined error bars<br>*State explicitly what error bars represent (e.g. SD, SE, CI)*

Our web collection on statistics for biologists may be useful.

Software and code

Policy information about availability of computer code

Data collection	No software was used for data collection.
Data analysis	Prism version 9.0 (GraphPad Software, San Diego, CA).<br>Past 3.20 (Natural History Museum, University of Oslo, Norway).

For manuscripts utilizing custom algorithms or software that are central to the research but not yet described in published literature, software must be made available to editors/reviewers upon request. We strongly encourage code deposition in a community repository (e.g. GitHub). See the Nature Research guidelines for submitting code & software for further information.

Data

Policy information about availability of data

All manuscripts must include a data availability statement. This statement should provide the following information, where applicable:

- Accession codes, unique identifiers, or web links for publicly available datasets
- A list of figures that have associated raw data
- A description of any restrictions on data availability

The authors declare that [the/all other] data supporting the findings of this study are available within the paper [and its supplementary information files].

Field-specific reporting

Please select the best fit for your research. If you are not sure, read the appropriate sections before making your selection.

- [x] Life sciences
- [ ] Behavioural & social sciences
- [ ] Ecological, evolutionary & environmental sciences

For a reference copy of the document with all sections, see nature.com/authors/policies/ReportingSummary-flat.pdf

Life sciences study design

All studies must disclose on these points even when the disclosure is negative.

Sample size	We performed pilot studies with n = 4 per treatment group. We found these sample sizes to be sufficient to detect differences in our primary outcome (hepatic triglyceride content). We then repeated animal cohorts using similar or larger sample sizes to validate our pilot findings.
Data exclusions	We pre-determined that any signs of illness, (e.g. weight loss, failed grooming, hunched/fluffed appearance, etc.), would preclude analysis of any given animal. However, no animals met this exclusion criteria
Replication	Attempts at replication were successful. e repeated each in vitro assay independently at least twice.
Randomization	All mice were randomly assigned to experimental groups.
Blinding	Investigators were not fully blinded during animal experiments. Investigators were always genotype blind. However, it is difficult to remain fully blind to dietary stimulus because mice fed a high-fat diet are visibly larger than chow-fed mice.

Reporting for specific materials, systems and methods

Materials & experimental systems

n/a	Involved in the study	
[x]	[ ]	Unique biological materials
[ ]	[x]	Antibodies
[ ]	[x]	Eukaryotic cell lines
[x]	[ ]	Palaeontology
[ ]	[x]	Animals and other organisms
[x]	[ ]	Human research participants

Methods

n/a	Involved in the study	
[x]	[ ]	ChIP-seq
[x]	[ ]	Flow cytometry
[x]	[ ]	MRI-based neuroimaging

Unique biological materials

Policy information about availability of materials

Obtaining unique materials	No unique material was used in this current study.

Antibodies

Antibodies used	Details on antibodies are provided in the Methods section. Antibodies against GAPDH (#5174), SIRT1 (#2028), and acetyl-lysine (#9441) were purchased from Cell Signaling Technology (CST, Beverly, MA, USA). NAMPT (#ab236874), UCP1 (#ab155117) and FGF21 (#ab171941) antibodies were obtained from Abcam (Cambridge, MA). The dilution for all primary antibodies was 1:1,000 in 5% non-fat milk in tris-buffered saline with tween 20 (TBST). Secondary antibodies were peroxidase-conjugated anti-rabbit and anti-mouse IgG (#7074S and #7076S respectively, CST, Beverly, MA, USA, 1:5,000-1:10,000 dilution).
Validation	The antibodies have been either validated in prior studies or validated with positive controls in the current study. GAPDH (Mayer, ..., DeBosch et al Sci Rep 2016), SIRT1 (Hong,..., and Pissios et al Nature Med 2015), acetyl-lysine (Stromsdorfer, ..., and Yoshino Cell Reports 2016) UCP1 (Zhao et al Mol Metab 2021) and FGF21 (Pereira, ..., and Abel eLife 2021) .

Eukaryotic cell lines

Policy information about cell lines

Cell line source(s)	Primary hepatocytes, isolated from male C57B/6 mice.
Authentication	not applicable (primary cells)
Mycoplasma contamination	We did not test for mycoplasma.

Commonly misidentified lines (See ICLAC register)	No commonly misidentified lines were used in this current study.

Animals and other organisms

Policy information about studies involving animals; ARRIVE guidelines recommended for reporting animal research

Laboratory animals	For overexpression experiments, C57B/6 males obtained directly from Jackson Laboratory, age 5-11wks of age were used in all studies, both for db/db studies and western diet fed studies. SIRT1 LKO mice were obtained directly from Jackson Laboratory and bred in-house. 5-11wk old male SIRT1 LKO mice were used for experiment. NAMPT LKO mice were obtained from Shin Imai, Washington University SOM. 5-11wk old male NAMPT LKO were used for experiment. Mice were housed at 23degC ambient temperature at 40-60% humidity.
Wild animals	The study did not involve wild animals.
Field-collected samples	The study did not involve samples collected from the field.
